# CXCL6 Reshapes Lipid Metabolism and Induces Neutrophil Extracellular Trap Formation in Cholangiocarcinoma Progression and Immunotherapy Resistance

**DOI:** 10.1002/advs.202503009

**Published:** 2025-04-30

**Authors:** Tian He, Zi‐Yi Wang, Bin Xu, Cheng‐Jie Zhong, Lu‐Na Wang, Huan‐Chen Shi, Zi‐Yue Yang, Shi‐Qi Zhou, Hui Li, Bo Hu, Xiao‐Dong Zhu, Ying‐Hao Shen, Jian Zhou, Jia Fan, Hui‐Chuan Sun, Cheng Huang

**Affiliations:** ^1^ Department of Hepatobiliary Surgery and Liver Transplantation Liver Cancer Institute and Zhongshan Hospital Fudan University Shanghai 200032 China

**Keywords:** Cholangiocarcinoma, CXCL6, Lipid metabolism, Neutrophil extracellular traps, Immunotherapy

## Abstract

The chemokine CXCL6 is identified as a pivotal regulator of biological processes across multiple malignancies. However, its function in cholangiocarcinoma (CCA) is underexplored. Tumor profiling for *CXCL6* is performed using a public database. Both in vitro and in vivo experiments are utilized to evaluate the oncogenic effects of CXCL6 on CCA. Additionally, RNA‐Seq is employed to detect transcriptomic changes related to CXCL6 expression in CCA cells and neutrophils. Molecular docking, fluorescence colocalization, and Co‐IP are used to elucidate a direct interaction between JAKs and CXCR1/2. Additionally, LC‐MS lipidomics and explored the impact of CXCL6 on immunotherapy in vivo. CXCL6 is upregulated in CCA tissues and promoted the proliferation and metastasis of CCA. Mechanistically, CXCL6 regulated the CXCR1/2‐JAK‐STAT/PI3K axis in CCA via autocrine signaling, leading to lipid metabolic reprogramming, and promoted neutrophil extracellular traps (NETs) formation by activating the RAS/MAPK pathway in neutrophils. Eventually, NETs formation induced immunotherapy resistance in CCA by blocking CD8^+^T cell infiltration. CXCL6 modulates CCA progression through the CXCR1/2‐JAK‐STAT/PI3K axis and reshaping its lipid metabolism. CXCL6 also mediates immunotherapy resistance through NETs, which may be a potential therapeutic target and biomarker for CCA.

## Graphical abstract

CXCL6's function in cholangiocarcinoma is underexplored. Here it is discovered that CXCL6 is upregulated in cholangiocarcinoma tissues and promotes proliferation and metastasis of it. CXCL6 functions through the CXCR1/2‐JAK‐STAT/PI3K axis and reshaping tumor lipid metabolism. CXCL6 also mediates immunotherapy resistance through NETs by blocking CD8^+^T cell infiltration, which may be a potential therapeutic target and biomarker for cholangiocarcinoma.

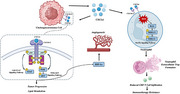

## 1 Introduction

The global incidence of CCA is rising, particularly in Asia.^[^ ^1^, ^2^ ^]^ CCA originates from bile duct epithelial cells and is usually further subdivided into intrahepatic cholangiocarcinoma (iCCA), periportal cholangiocarcinoma (pCCA), and distal cholangiocarcinoma (dCCA).^[^ ^3^ ^]^ Due to its insidious onset, most patients are diagnosed at the advanced stage, with only 15–30% eligible for surgery and the recurrence rate exceeding 50%.^[^ ^4^ ^]^

This being the case, systemic drug therapy has become the mainstay for advanced CCA. Chemotherapy based on gemcitabine (GEM) was traditionally the most common treatment,^[^ ^5^, ^6^ ^]^ although advances in sequencing technology enabled mutated genes related to CCA to be systematically analyzed, inspiring a wave of targeted treatments. Drugs targeting FGFR2, IDH1/2, HER2, and IDH2 have been widely reported and even put into clinical use in CCA.^[^ ^7^ ^]^ Meanwhile, combining chemotherapy with immunotherapy opened up a new era, such that GEM plus cisplatin (GC) in combination with immunotherapy has now become the first‐line treatment for CCA.^[^ ^8^ ^]^ Nevertheless, the latest results from the TOPAZ‐1 clinical trial indicate an overall response rate of only 26.7% with GC plus the anti‐PD‐L1 antibody durvalumab, which is still far from satisfactory.^[^ ^9^ ^]^ Further research is needed to understand the tumor immune microenvironment of CCA and identify suitable strategies to overcome immune resistance.

The TIME of CCA is a highly complex, dynamic system comprising tumor cells, immune components, and blood vessels, among other constituents.^[^ ^10^ ^]^ These different components interact with each other in ways that greatly affect the occurrence, development, and immune resistance of CCA,^[^ ^11^ ^]^ which is characterized as a “cold tumor” with dense desmoplastic stroma that may secrete angiogenesis‐promoting molecules such as vascular endothelial growth factors (VEGF).^[^ ^12^ ^]^ Thus, targeting angiogenesis is a potential anti‐CCA option and the entry point of our study.

Immune cells are also considered functionally important in mediating CCA immunotherapy resistance. As well as CD8^+^ T cells, which have been extensively studied, innate immune cells like tumor‐associated neutrophils (TANs) have also been shown to be closely related to tumor development and overall survival (OS) of patients.^[^ ^13^ ^]^ The number of TANs has been associated with the prognosis of CCA surgery patients but the functional mechanisms have not been fully analyzed.^[^ ^14^ ^]^ However, recent research revealed that TANs can form NETs in the extracellular space and greatly reshape the TIME,^[^ ^15^ ^]^ suggesting an important way in which TANs might induce immunotherapy resistance in CCA.

Furthermore, chemokines may function as the bridge between CCA and neutrophils. The C‐X‐C motif chemokine ligand (CXCL) subfamily is an important class of chemokines with close connections to tumor biological processes;^[^ ^16^ ^]^ CXCLs act as chemotactic factors for TANs, mediating their recruitment into the TIME to promote tumor drug resistance.^[^ ^17^ ^]^ These chemokines also directly regulate processes in cancer cells. For example, CXCL1, CXCL2, and CXCL8 reportedly affect the proliferation of lung cancer cells and also influence angiogenesis, which may lead to tumor infiltration of neutrophils and macrophages and induce anlotinib resistance.^[^ ^18^, ^19^ ^]^

By binding to C‐X‐C motif chemokine receptors (CXCRs), CXCLs activate several tumor‐associated pathways including MAPK, STAT, TGF‐β, and β‐catenin, thereby modulating the development and metabolism of tumor cells,^[^ ^20^, ^21^ ^]^ hinting at a possible mechanistic link between chemokine signaling and CCA treatment resistance. Notably, overexpression of CXCL6 was reported in high‐stemness malignant CCA cells;^[^ ^22^ ^]^ it was also found to promote tumor progression in hepatocellular carcinoma (HCC) and non‐small cell lung cancer,^[^ ^23^, ^24^ ^]^ and to regulate neutrophil infiltration in gastric cancer.^[^ ^25^ ^]^ However, the function and mechanism of CXCL6 in CCA have not been investigated in detail.

Here, we aimed to explore the functions and mechanisms of CXCL6 in CCA cells and the TIME. We demonstrated that expression of CXCL6 regulated proliferation, metastasis, and prognosis in CCA through modulating the CXCR1/2‐JAK‐STAT/PI3K axis. Eventually, CXCL6 could reshape lipid metabolism in CCA cells, and its overexpression also related to CCA immunotherapy resistance since CXCL6 induced NETs formation and led to reduced infiltration of CD8^+^ T cells. Overall, our results position CXCL6 as a pivotal regulator in CCA development and immunotherapy resistance by reshaping lipid metabolism and inducing the formation of NETs.

## 2 Results

### 2.1 CXCL6 Is Upregulated in CCA Tumor Tissue and Correlates with Poor Prognosis and Enhanced Malignancy

**Figure 1:**
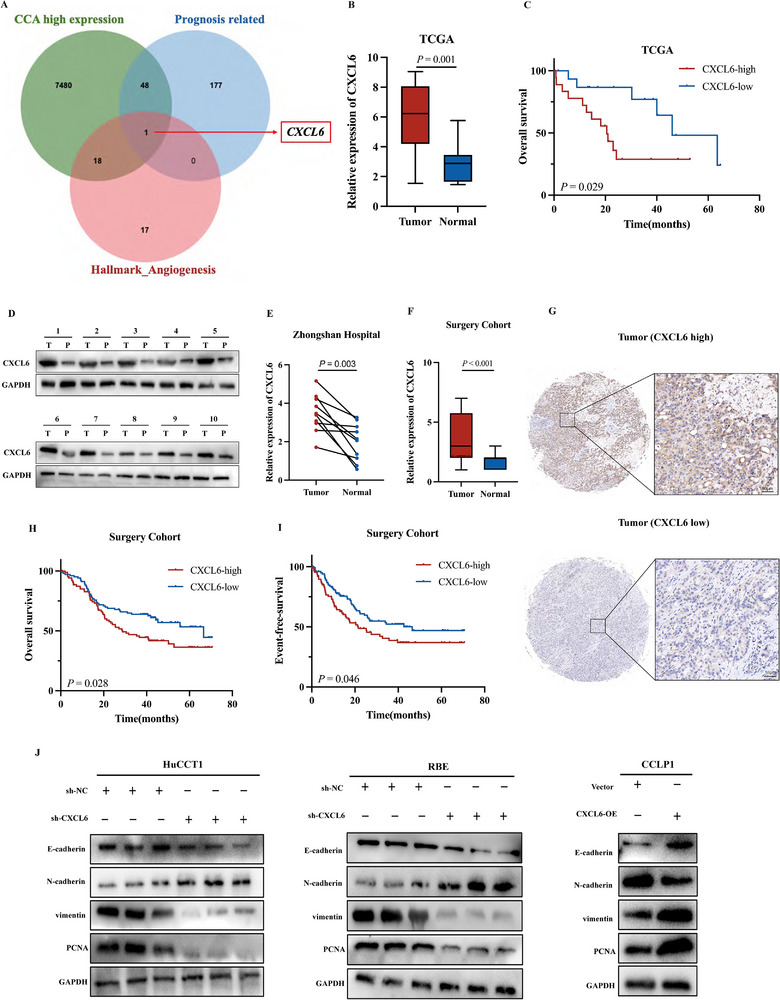
CXCL6 is upregulated in CCA tumor tissue and relates to poor prognosis and greater malignancy. A) Identification of *CXCL6* based on TCGA data. B) *CXCL6* is highly expressed in tumor tissue, based on TCGA. C) High expression of *CXCL6* is associated with poorer OS, based on TCGA. D) Western blot analysis of CXCL6 expression in CCA tumor and paired non‐tumor tissues. E) RT‐qPCR analysis of *CXCL6* expression in CCA tumor and paired non‐tumor tissues. F) Comparison of CXCL6 expression in tumor and paired non‐tumor tissues in the “Surgery cohort” TMA. G) Representative images of high/low CXCL6 expression specimens in the “Surgery cohort” TMA; scores were calculated based on the intensity and percentage of stained cells. H,I) Upregulation of CXCL6 in CCA was correlated with worse OS and EFS in the “Surgery cohort” TMA. J) Western blot analysis showing that knocking‐down or overexpression of CXCL6 affects the expression of E‐cadherin, N‐cadherin, vimentin, and PCNA in CCA cells.

Angiogenesis‐related checkpoints are considered to be major drivers of CCA progression and development.^[^ ^26^ ^]^ Therefore, in addition to containing vascularization, antiangiogenic agents may remodel the TIME and potentiate the efficacy of chemotherapy and immunotherapy.^[^ ^27^ ^]^ To identify key genes contributing to angiogenesis and progression in CCA, we explored The Cancer Genome Atlas (TCGA) database (TCGA‐CHOL) to look for the intersection of genes that were highly expressed in CCA (log_2_|FC| ≥ 1, *P* ≤ 0.05), prognosis‐related (HR > 1, *P *≤ 0.1), and associated with angiogenesis (i.e., genes belonging to the “HALLMARK_ANGIOGENESIS” dataset from the Molecular Signatures Database). *CXCL6* was the sole gene fulfilling all criteria (**Figure** **1**A–C; Figure S1A, Supporting Information) and was further validated as a prognostic marker for DSS in CCA (Figure S1B, Supporting Information).

Pan‐cancer analysis based on TCGA data revealed elevated *CXCL6* expression across multiple malignancies, especially in CCA (*P* ≤ 0.01, Figure S1C, Supporting Information). We corroborated this finding by comparing 10 paired surgical resection tissue specimens from CCA patients in Zhongshan Hospital. Western blot and RT‐qPCR results showed elevated CXCL6 levels in tumor tissues versus paired non‐tumor tissue (Figure 1D,E). Differential tumor expression was further confirmed by IHC staining of the “Surgery cohort” tissue microarray (TMA) from Zhongshan Hospital, allowing us to reach the same conclusion that CXCL6 is overexpressed in tumor tissue (*P* < 0.001, Figure 1F). Using the same stained TMA to analyze the clinical significance of CXCL6 (Figure 1G) revealed that patients with higher expression had significantly worse OS (*P* = 0.028, Figure 1H) and EFS (*P* = 0.046, Figure 1I).

To explore its effects on tumorigenesis at the cellular level, we first quantified CXCL6 protein levels in a panel of five common CCA cell lines (Figure S1D, Supporting Information). HuCCT1 and RBE cells exhibited the highest CXCL6 expression, whereas CCLP1 showed the lowest. Thus, we chose to knock down *CXCL6* in HuCCT1 and RBE cell lines and overexpress it in CCLP1 cells. We established sh‐CXCL6 (*CXCL6* knockdown via three targeting and one nontargeting control [NC] shRNAs) and CXCL6‐OE (*CXCL6* overexpression via plasmids) cell lines and confirmed the transfection efficiency of lentivirus and plasmid by western blot (Figure S1E, Supporting Information). Western blot analysis of knockdown and overexpression cell lines revealed that CXCL6 is associated with metastasis (E‐cadherin and N‐cadherin), invasion (vimentin), and proliferation (PCNA) in CCA (Figure 1J).

### 2.2 CXCL6 Promotes CCA Development and Sensitizes Gemcitabine Effects Both In Vitro and In Vivo

**Figure 2:**
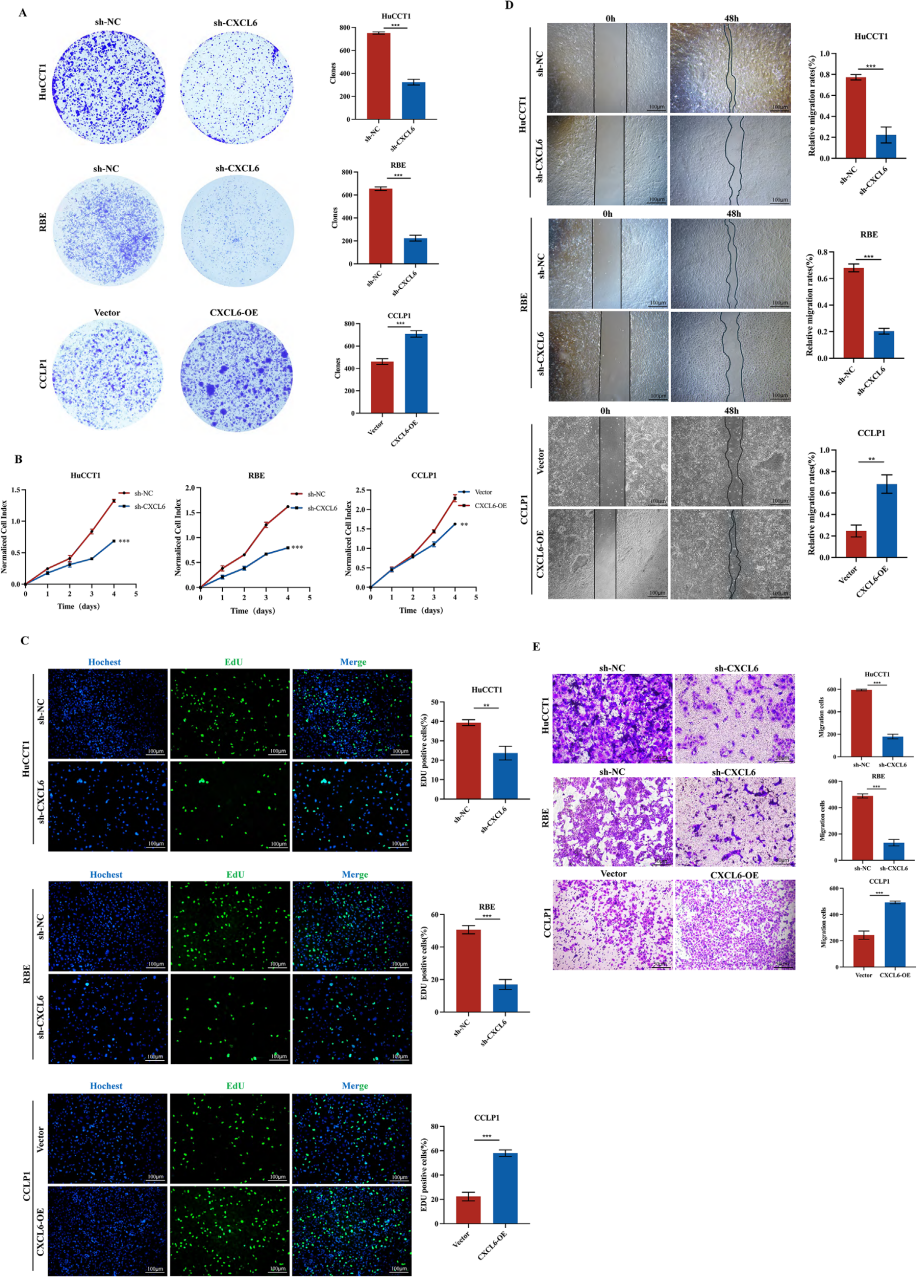
CXCL6 promotes CCA proliferation, migration, and angiogenesis in vitro. A) Colony formation assay with HuCCT1, RBE, and CCLP1 cell lines. B,C) CCK‐8 and EdU proliferation assays were used to assess the effect of CXCL6 overexpression. D,E) Wound healing and transwell assays indicated that CXCL6 promotes metastasis of CCA cells.

We next conducted functional assays in vitro to evaluate the role of CXCL6 in cellular proliferation and CCA development. Silencing *CXCL6* impaired the clonogenic growth of HuCCT1 (*P* < 0.001) and RBE (*P* < 0.001) cells, while *CXCL6* overexpression increased the colony formation of CCLP1 cells (*P* < 0.001; **Figure** **2**A). CCK‐8 cell proliferation experiments showed the same trend (Figure 2B), as did EdU incorporation assays to verify the impact of *CXCL6* knockdown or overexpression on cellular proliferation (Figure 2C). Wound healing and transwell assays with the sh‐CXCL6 and CXCL6‐OE lines jointly demonstrated that CXCL6 has positive effects on the migration of CCA cells (Figure 2D,E). To investigate its association with angiogenesis, we collected conditioned medium from CCA cell cultures and used this to culture HUVECs in a tube formation assay. ELISA confirmed reduced CXCL6 levels in sh‐*CXCL6* supernatants and elevated levels in CXCL6‐OE supernatants (Figure S2A–C, Supporting Information). When cultured with sh‐CXCL6 conditioned medium, the tube formation capacity of HUVECs was significantly attenuated, whereas it was enhanced in the presence of CXCL6‐OE conditioned medium (Figure S2D, Supporting Information).

**Figure 3:**
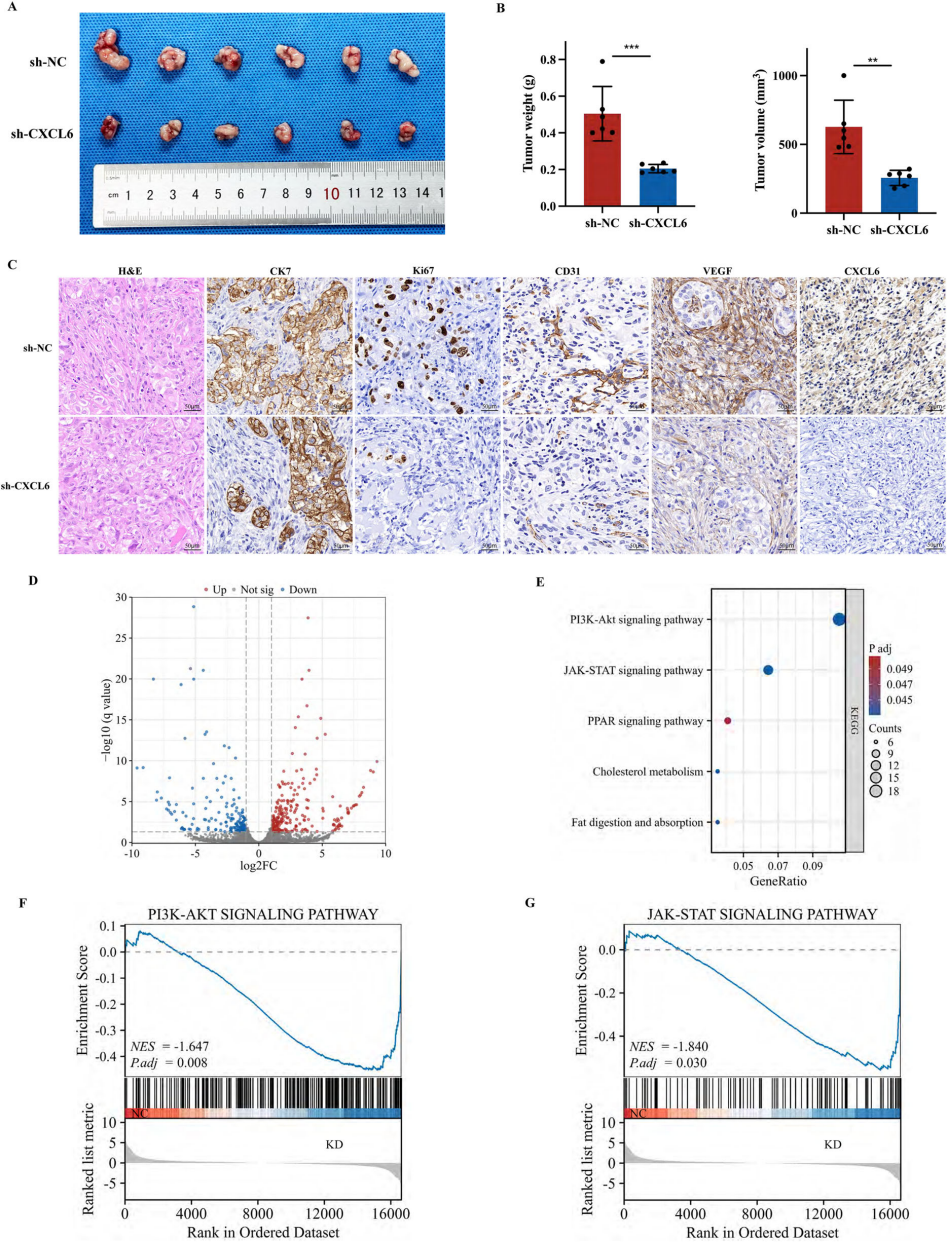
CXCL6 regulates CCA proliferation and migration through the JAK‐STAT/PI3K‐AKT pathway. A,B) Representative images and quantification of subcutaneous HuCCT1 xenograft tumors established in BALB/c nude mice, with or without *CXCL6* knockdown. C) Expression levels of CK7, Ki67, CD31, VEGF, and CXCL6 in xenograft tumors were determined by IHC staining. D) Volcano plot of differential gene expression between sh‐NC and sh‐CXCL6 HuCCT1 cells. E) Results of KEGG pathway enrichment analysis based on RNA‐Seq of CCA cells (i.e., sh‐CXCL6 vs sh‐NC HuCCT1 cells). F,G) GSEA results based on the RNA‐Seq data.

We subsequently performed in vivo analysis in BALB/c nude mice to assess the function of CXCL6, using sh‐NC or sh‐CXCL6 HuCCT1 cells to focus specifically on its impact on tumor cells. The results showed that CXCL6 depletion significantly reduced tumor weight (*P* < 0.001) and volume (*P* = 0.001; **Figure** **3**A,B; Figure S3A, Supporting Information). H&E and IHC staining confirmed decreased malignancy (Ki‐67) and angiogenesis (CD31, VEGF) in sh‐*CXCL6* tumors (Figure 3C). The above results together illustrate that CXCL6 may significantly impact the malignancy, proliferation, and angiogenesis in CCA tumors.

Since GEM‐based chemotherapy is still a first‐line choice in unresectable CCA,^[^ ^28^ ^]^ we next asked whether CXCL6 could modulate its therapeutic efficacy. In vitro, *CXCL6* knockdown synergized with GEM to suppress proliferation (Figure S3B,C, Supporting Information) and migration (Figure S3D,E, Supporting Information) of CCA cells. Treating CCA cell lines with 200 nm GEM indicated (via γ‐H2AX assay) that *CXCL6* knockdown aggravated drug‐induced DNA damage in both HuCCT1 and RBE cells (Figure S4A, Supporting Information). This sensitization effect of *CXCL6* knockdown was confirmed in vivo by establishing a mouse xenograft model. As before, sh‐NC and sh‐CXCL6 HuCCT1 cells were injected subcutaneously in BALB/c nude mice, and details of the dosing regimen are shown in Figure S4B (Supporting Information). While GEM treatment clearly reduced tumor weight and tumor volume, silencing of *CXCL6* further enhanced its efficacy (Figure S4C–E, Supporting Information). Taken together, the foregoing results illustrate that silencing *CXCL6* may overcome GEM resistance in CCA.

### 2.3 CXCL6 Regulates CCA Proliferation and Migration through the JAK‐STAT/PI3K‐AKT Pathway

To explore potential mechanisms underlying CXCL6‐mediated regulate proliferation and migration in CCA, RNA‐Seq was performed on sh‐NC and sh‐CXCL6 HuCCT1 cells, identifying 233 significantly upregulated and 171 significantly downregulated genes (with *q*‐value ≤ 0.05 and log|FC| ≥ 1; Figure 3D). Applying KEGG pathway analysis identified five significantly enriched pathways, among which the PI3K‐AKT and JAK‐STAT pathways were most significantly enriched in sh‐CXCL6 cells versus sh‐NC cells (Figure 3E). GSEA similarly highlighted significant enrichment of the “PI3K‐AKT signaling pathway” and “JAK‐STAT signaling pathway” (Figure 3F,G). The JAK‐STAT pathway, activated by cytokines,^[^ ^29^ ^]^ has been implicated in carcinogenesis and development in multiple tumors^[^ ^30^, ^31^ ^]^ and is also able to regulate bile duct cell proliferation and function.^[^ ^32^ ^]^ Similarly, PI3K‐AKT is a prominent signal transduction pathway activated in both solid and hematologic tumors.^[^ ^33^ ^]^ In CCA, this pathway is known to play a central role in proliferation, cell cycle regulation, and metabolism.^[^ ^34^ ^]^ We therefore assumed that CXCL6 may exert its effects in CCA through JAK‐STAT and PI3K‐AKT signaling.

To validate the pathway enrichment results, we applied western blot with sh‐CXCL6 HuCCT1 and RBE cells and CXCL6‐OE CCLP1 cells to detect crucial factors in both pathways, observing reduced activation with *CXCL6* knockdown and increased pathway activity upon *CXCL6* overexpression (Figure S5A,B, Supporting Information). To seek further confirmation, cells were treated with the JAK1/2 inhibitor ruxolitinib or the PI3K inhibitor 3‐methyladenine (3‐MA). While CXCL6 overexpression enhanced in vitro migration (Figure S6A,B, Supporting Information) and proliferation (Figure S6C–E, Supporting Information) in these cells, both effects were weakened by the addition of ruxolitinib or 3‐MA. These data supported the involvement of the JAK‐STAT and PI3K‐AKT pathways in CXCL6 oncogenic function.

### 2.4 CXCL6 Activates the CXCR1/2‐JAK‐STAT/PI3K Axis in CCA

We further elucidated how CXCL6 regulates JAK‐STAT and PI3K‐AKT signaling in CCA by considering that CXCL6, as a secretory chemokine, must bind to its membrane surface receptors CXCR1/2 to regulate cellular functions.^[^ ^35^ ^]^ Expression of both receptors has been reported on neutrophils, endothelial cells, and tumor cells,^[^ ^36^, ^37^ ^]^ where they are involved in neutrophil recruitment, inflammatory disease, and tumor progression.^[^ ^37^, ^38^, ^39^ ^]^ The JAK family proteins can bind to and activate cytokine receptors by phosphorylating those receptors upon ligand binding, leading to the formation of dimeric Janus kinase cytokine receptor complexes^[^ ^40^ ^]^ that may further recruit and phosphorylate proteins like STAT or PI3K.^[^ ^41^, ^42^ ^]^ Based on this, we hypothesized that in CCA cells, CXCL6 might exert its observed effects through the CXCR1/2‐JAK‐STAT/PI3K axis. While the binding of JAKs to CXCR1/2 has not previously been proven, we conducted the following experiments to test our hypothesis.

**Figure 4:**
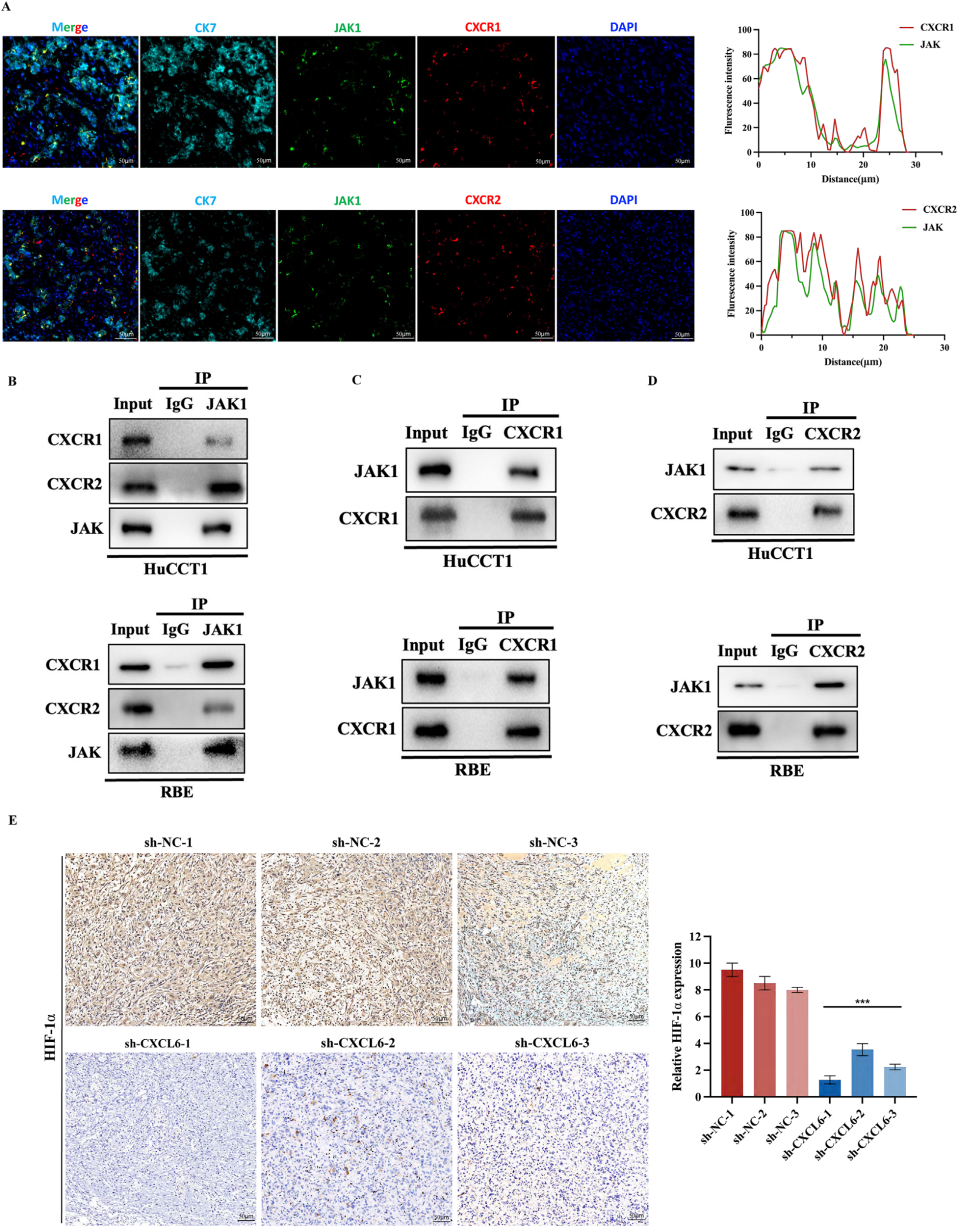
CXCL6 functions through activating the CXCR1/2‐JAK‐STAT/PI3K axis in CCA. A) Representative images and analysis plots from immunofluorescence experiments, demonstrating colocalization of CXCR1/JAK1 and CXCR2/JAK1 in CCA tissue. B) Co‐IP results using JAK1 as bait indicated interactions between JAK1/CXCR1 and JAK1/CXCR2. C,D) Co‐IP results using CXCR1/2 as bait supported CXCR1/JAK1 and CXCR2/JAK1 interactions in HuCCT1 and RBE cells. E) HIF‐1α IHC staining of nude mouse tumor tissue.

Immunofluorescence staining in paraffin‐embedded CCA tissue revealed strong spatial colocalization of JAK1/CXCR1 and JAK1/CXCR2 (**Figure** **4**A). Co‐IP assays with JAK1 as bait revealed interactions with CXCR1 and CXCR2 in HuCCT1 and RBE cells (Figure 4B). Reciprocal Co‐IP with CXCR1/2 as bait further validated these interactions (Figure 4C,D). We also applied molecular docking to evaluate possible binding between JAK1 and the CXCL6 receptors. Using GRAMM,^[^ ^43^ ^]^ we conducted semiflexible docking between JAK1/CXCR1 and JAK1/CXCR2; the results showed stable predicted binding of JAK1 to both receptors (Figure S7A,B, Supporting Information). The computed JAK1/CXCR1 binding energy was −35.1 kcal mol^−1^ and the interface area was 3523 Å^2^ (Figure S7C, Supporting Information), while the equivalent values for JAK1/CXCR2 were −8.1 kcal mol^−1^ and 3130.9 Å^2^, respectively (Figure S7D, Supporting Information). Overall, the above results indicate the binding of JAK1 to both CXCL6 receptors. To functionally validate receptor involvement, we employed reparixin and SB225002 as selective antagonists for CXCR1 and CXCR2, respectively.^[^ ^44^, ^45^ ^]^ Treatment with either inhibitor impaired phosphorylation of JAK (Figure S7E, Supporting Information). In CXCL6‐OE CCLP1 cells, reparixin and SB225002 were able to inhibit malignancy (Figure S7F, Supporting Information), proliferation (Figure S7G–I, Supporting Information), and migration (Figure S7J,K, Supporting Information).

Since it was reported that the JAK‐STAT and PI3K‐AKT pathways both activate HIF‐1α expression,^[^ ^46^, ^47^ ^]^ we applied IHC staining in nude mouse tumors and found that HIF‐1α expression was lower in sh‐CXCL6 cells (Figure 4E). In conclusion, the CXCR1/2‐JAK‐STAT/PI3K axis is a potential mechanism through which CXCL6 plays its role in CCA.

### 2.5 CXCL6 Reshapes CCA Lipid Metabolism

**Figure 5:**
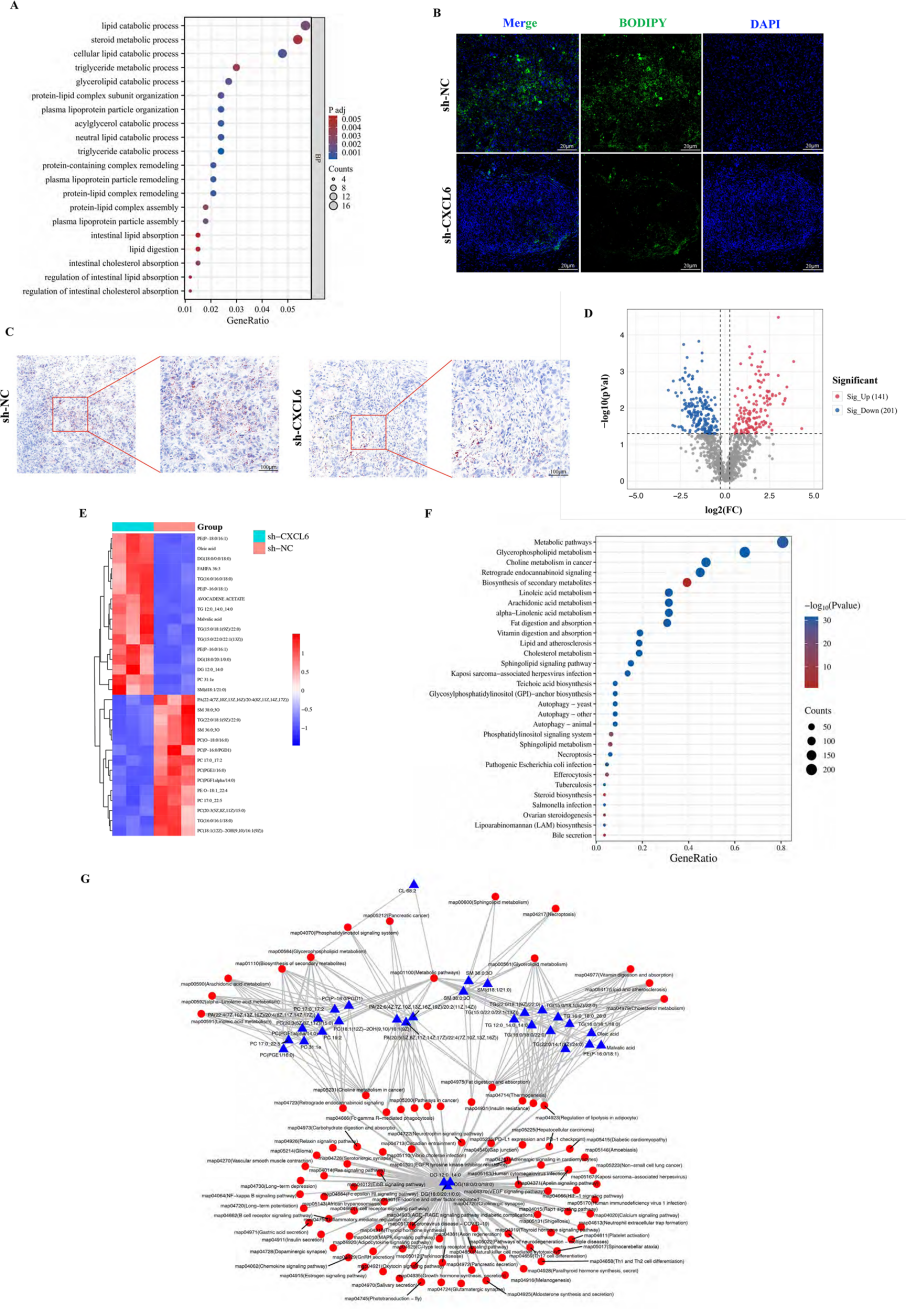
CXCL6 reshapes CCA lipid metabolism. A) GO pathway enrichment of RNA‐Seq data from CCA cells (sh‐NC vs sh‐CXCL6 HuCCT1 cells). B,C) BODIPY and Oil Red O staining of murine xenograft CCA tumors. D) Volcano plot of differential metabolites in LC‐MS lipidomics. E) Heat map of the top 30 differential metabolites (sh‐NC vs sh‐CXCL6 HuCCT1 cells). F) KEGG analysis based on the top 30 differential metabolites. G) Network of these metabolites and their association with cellular pathways.

The JAK‐STAT and PI3K‐AKT pathways regulate biological processes in tumor cells in various ways.^[^ ^48^, ^49^ ^]^ Notably, the JAK‐STAT pathway was shown to boost de novo fatty acid synthesis and fatty acid uptake in the TIME,^[^ ^48^, ^50^ ^]^ while PI3K‐AKT activation can cause lipid peroxidation in gastric cancer.^[^ ^51^ ^]^ To explore these possibilities in CCA, we applied GO‐BP (Gene Ontology – Biological Processes) analysis to the RNA‐Seq data mentioned above and noted that lipid metabolism‐related pathways were most highly enriched (**Figure** **5**A). Subsequent BODIPY and Oil Red O staining of murine CCA xenografts demonstrated that *CXCL6* knockdown markedly reduced intratumoral lipid accumulation (Figure 5B,C), suggesting CXCL6‐driven lipid metabolic remodeling

Applying LC–MS lipidomics to analyze sh‐NC and sh‐CXCL6 HuCCT1 cells allowed us to identify 342 differential metabolites (Figure 5D). We listed the top 30 metabolites with the largest differences and found that in sh‐CXCL6 cells, the most downregulated metabolites belonged to the phosphatidylcholine class (Figure 5E); phosphatidylcholines are reported to promote tumor cell proliferation and impair CD8^+^ T cell function.^[^ ^52^, ^53^ ^]^ KEGG pathway analysis of these metabolites highlighted enrichment in cancer‐associated lipid pathways, including “Glycerophospholipid metabolism,” “Linoleic acid metabolism,” and “Arachidonic acid metabolism”^[^ ^54^, ^55^, ^56^ ^]^ (Figure 5F). Constructing a network revealed that “DG 12:0/14:0,” “DG (18:0/20:1/0:0),” and “DG (18:0/0:0/18:0)” were linked to the most pathways (Figure 5G). Importantly, diacylglycerol (DG) is known as a double‐edged sword affecting tumor progression and can also regulate the TIME by activating CD8^+^ T cells and enhancing the cytotoxicity of natural killer (NK) cells.^[^ ^57^, ^58^ ^]^ In sum, CXCL6 may significantly affect the content of key metabolites in CCA, which can further regulate tumor progression and TIME in return.

### 2.6 CXCL6 Expression Correlates with Immunotherapy Resistance and TAN Infiltration in CCA

**Figure 6:**
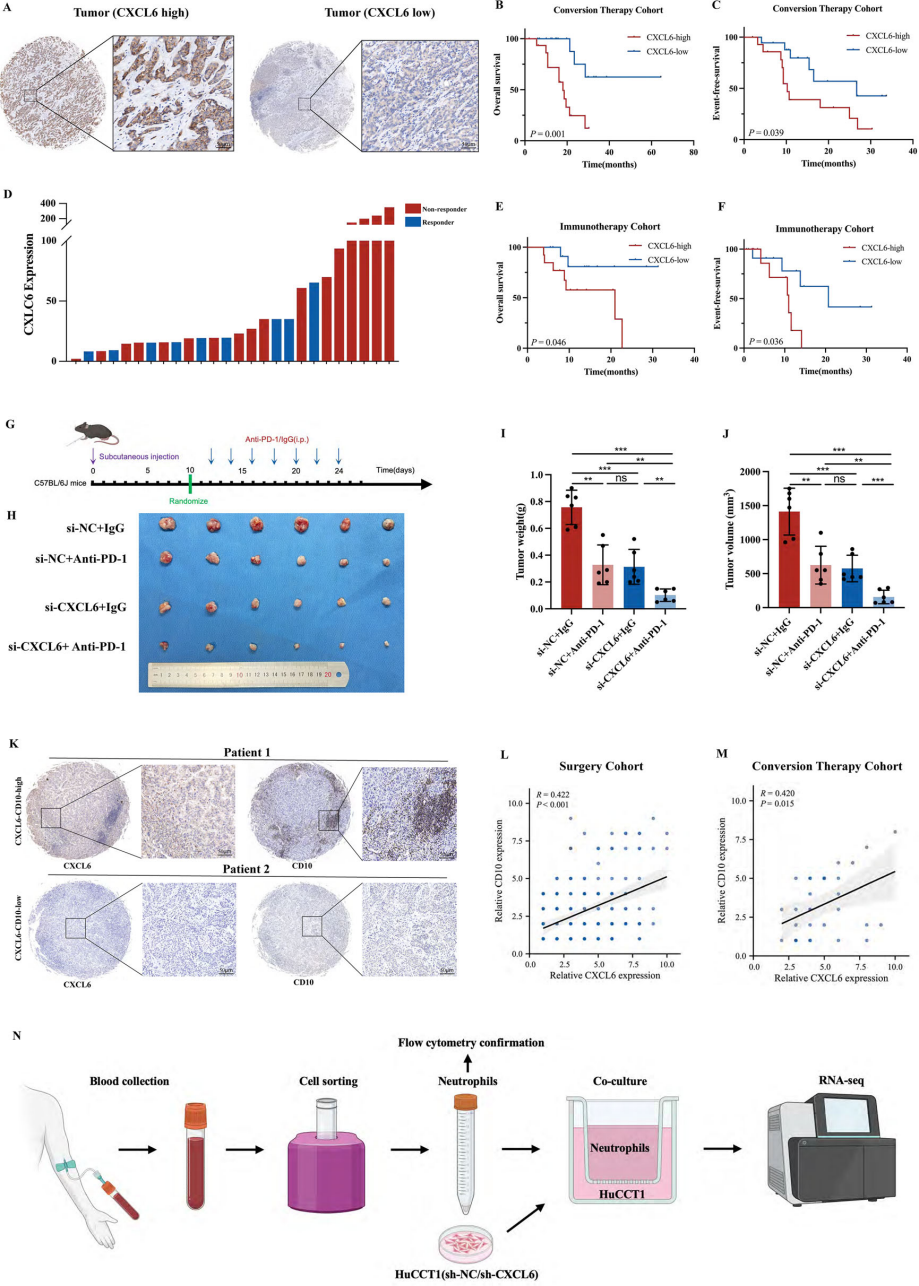
CXCL6 expression is associated with CCA immunotherapy resistance and TAN infiltration level. A) Representative images of CXCL6 IHC staining in the “Conversion therapy cohort” TMA. B,C) OS, EFS analysis. D) ELISA measurements of CXCL6 in baseline peripheral serum. E,F) Comparison of OS and EFS between CXCL6‐high and CXCL6‐low patients. G) Experimental design and dosing regimen for the C57BL/6J mouse subcutaneous tumors. H–J) Representative images of subcutaneous tumors along with quantification of tumor weight and volume. K) Representative images of CXCL6 and CD10 IHC staining in the TMA. L–M) Linear regression analysis of CXCL6 and CD10 expression in the “Surgery cohort” and “Conversion therapy cohort” TMAs. N) Workflow for neutrophil isolation, coculture, and RNA‐Seq analysis.

The above results indicate that CXCL6 exerts direct regulation on CCA cells and could therefore affect GEM treatment sensitivity, leading us to ask whether CXCL6 expression levels are associated with CCA immunotherapy response. Through IHC staining of the “Conversion therapy cohort” TMA, we found that CXCL6‐low patients (50% cutoff) benefited more from immunotherapy, experiencing longer OS and EFS (**Figure** **6**A–C). After collecting baseline peripheral blood from 26 CCA patients prior to immunotherapy (Table S3, Supporting Information), serum CXCL6 levels were measured by ELISA and showed that nonresponders tended to have higher serum concentrations of CXCL6 (Figure 6D). Higher CXCL6 levels were also associated with shorter OS and EFS after immunotherapy (Figure 6E,F). An animal subcutaneous xenograft study based on CCA cells of murine origin (mIC‐23) was conducted to corroborate the clinical findings, in C57BL/6J mice according to the experimental design shown in Figure 6G. *CXCL6* expression was knocking down using siRNAs, while si‐CXCL6‐3 was chosen for in vivo experiment (Figure S8A, Supporting Information). Silencing of *CXCL6* boosted the efficacy of anti‐PD‐1 therapy, significantly reducing tumor weight and volume (Figure 6H–J; Figure S8B, Supporting Information).

All in all, these results strongly indicate that CXCL6 may lead to CCA immunotherapy resistance, so we further explored the potential mechanisms. Specifically, CXCR1/2 is expressed on TANs where they regulate TIME recruitment and NETs formation.^[^ ^59^, ^60^ ^]^ Considering that CXCL6 is a chemokine that binds to these receptors, we studied its influence on TANs, applying IHC staining to detect the relationship between CXCL6 and TANs (CD10^+^) infiltration in two CCA TMAs. In both the “Surgery” and “Conversion therapy” cohorts, CXCL6 protein levels were positively associated with TANs density (*R* = 0.422, *P* < 0.001; *R* = 0.420, *P* = 0.015, Figure 6K–M). We also analyzed the correlation of CXCL6 with infiltration of other immune cells (T cells, macrophages, and Tregs), but no significant relationship was apparent in any case (Figure S8C–G, Supporting Information).

### 2.7 CXCL6 Promotes NETs Formation and Impairs CD8^+^ T Cell Infiltration

To explore the effects of CXCL6 on neutrophils, peripheral neutrophils were isolated from blood and cocultured with sh‐NC or sh‐CXCL6 HuCCT1 cells (Figure 6N); TANs were identified using flow cytometry as previously reported^[^ ^61^ ^]^ (Figure S8H, Supporting Information). By RNA‐Seq, we then identified 240 differently expressed genes (with log_2_|FC| ≥ 1, *q*‐value ≤ 0.05) between TANs cultured at the two different concentrations of CXCL6 (Figure S9A, Supporting Information).

**Figure 7:**
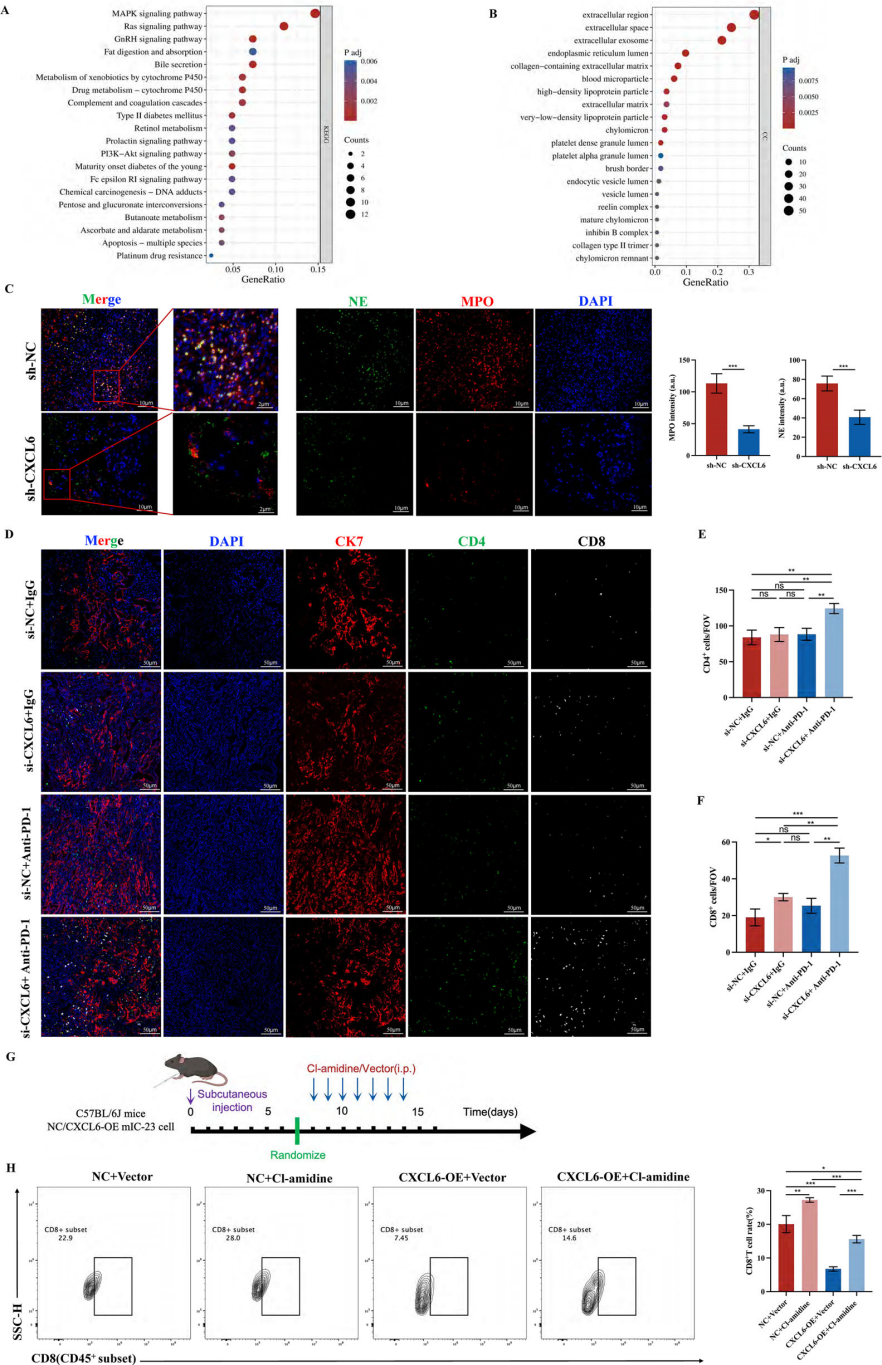
CXCL6 promotes NETs formation and inhibits CD8^+^ T cell infiltration in the TIME. A) KEGG enrichment analysis of significantly differentially expressed genes in TANs cocultured with sh‐NC versus sh‐CXCL6 HuCCT1 CCA cells. B) GO enrichment analysis of the same differentially expressed genes. C) mIHC staining of two NETs markers, neutrophil elastase (NE) and myeloperoxidase (MPO), in murine C57BL/6J tumors. D–F) mIHC staining for CD4, CD8, and CK7 in murine C57BL/6J tumors and statistical analysis. G) Experimental design and dosing regimen for the rescue experiment. H) Flow cytometry of CD8^+^CD45^+^ in rescue experiment.

Applying KEGG analysis showed that the “MAPK signaling pathway” and “Ras signaling pathway” ranked highest among differentially expressed genes (**Figure** **7**A), while GO analysis revealed “Extracellular region” and “Extracellular space” as the two highest‐ranked “Cellular component” terms (Figure 7B). Activation of the RAS/MAPK pathway in CXCL6‐exposed neutrophils was verified by western blot (Figure S9B, Supporting Information). This pathway was reported to be a key upstream regulator of NETs formation,^[^ ^62^, ^63^ ^]^ and CXCR1/2 are apparently also involved.^[^ ^64^ ^]^ Thus, our results strongly suggested a role for CXCL6 in NETs formation derived from TANs. mIHC staining of murine mIC‐23 tumors revealed that *CXCL6* knockdown greatly reduced the formation of NETs (Figure 7C), confirming this as a potential relevant mechanism. Considering that the generation of reactive oxygen species (ROS) is the key starting point for NET formation,^[^ ^65^ ^]^ we measured ROS levels in TANs after coculturing with HuCCT1 cells and found that ROS levels decreased in sh‐CXCL6 groups (Figure S9C,D, Supporting Information). Taken together, the above evidence reveals that CXCL6 may mediate the formation of NETs in the CCA TIME.

A previous study illustrated how NETs contribute to blockage of tumor T cell infiltration,^[^ ^66^ ^]^ so we also analyzed T cell populations in murine C57BL/6J tumors by mIHC staining (Figure 7D). Analysis of the data showed no significant difference in CD4^+^ T cell between “si‐NC+IgG”, “si‐CXCL6+IgG” and ‘si‐NC+Anti‐PD‐1′ groups (Figure 7E), while ‘si‐CXCL6+Anti‐PD‐1′ showed an increase. As for CD8^+^ T cell, knocking down of CXCL6 and anti‐PD‐1 treatment slightly increased the number of CD8^+^ T cell, the combination treatment showed the strongest recruitment of CD8^+^ T cell (Figure 7F). Since CD8^+^ T cell exhaustion reportedly causes immunotherapy resistance in CCA,^[^ ^67^ ^]^ we concluded that CXCL6 may underlie this resistance by reducing CD8^+^ T cell infiltration through NETs formation.

To further confirm the interaction between NETs, formation and CD8^+^ T cell infiltration, we conducted a rescue experiment as depicted in Figure 7G. Cl‐amidine was chosen to weaken the formation of NETs as reported.^[^ ^68^, ^69^ ^]^ which was a direct inhibitor of PAD4, a key inducer of NETs.^[^ ^70^ ^]^ Cl‐amidine reversed CXCL6‐driven tumor growth (Figure S9E, Supporting Information). Based on flow cytometry results, we found that the overexpression of CXCL6 greatly rose the formation of NETs, shown by ROS levels (Figure S9F, Supporting Information). At the meantime, the infiltration numbers of CD8^+^T cell were significantly reduced (Figure 7H). It was more persuasive that Cl‐amidine could reverse the effects of CXCL6 on NETs, as well as restore the infiltration of CD8^+^T cell. We also examined the IFNγ (Figure S9G, Supporting Information) and GzmB (Figure S9H, Supporting Information) levels in CD8^+^T cell, results showed that NETs could reduced the killing effects of CD8^+^T cell. The same conclusion could be observed from the mIHC staining of MPO, NE, and CD8 based on the same samples of tumor tissue (Figure S10A, Supporting Information). In all, the above results together show that CXCL6 induces the formation of NETs in CCA, which blocks the infiltration and killing function of CD8^+^T cell and eventually causes immunotherapy resistance.

## 3 Discussion

Accumulating studies suggest the involvement of the CXCLs cytokine family in the progression of multiple malignancies.^[^ ^71^ ^]^ As secreted factors, CXCLs can target a variety of cell types to exert different functions, and their roles have been particularly studied in HCC. For instance, Wu et al. reported that *CXCL6* overexpression in HCC cells activates the JAK‐STAT3 pathway in hepatocytes through paracrine signaling.^[^ ^72^ ^]^ Among liver cancers, the incidence of CCA is second only to HCC, yet the molecular mechanisms of CXCLs in CCA have barely been explored.

Our study identifies CXCL6 as a multifunctional oncoprotein in CCA. First, we discovered CXCL6 as a pro‐angiogenic factor, observing that it can raise VEGF levels in CCA (Figure 3C) and increase HIF‐1α expression (Figure 4E). As well as inducing a hypoxic environment, HIF‐1α acts as an upstream regulator of vascular growth factors.^[^ ^73^ ^]^ Second, we found that high CXCL6 expression in CCA cells accelerates tumor progression and metastasis through autocrine mechanisms. We also tested whether *CXCL6* silencing could sensitize cells to GEM, given that this is still a first‐line therapy for CCA. In vivo and in vitro experiments showed that GEM had stronger cytotoxic effects on CXCL6‐depleted CCA cells (Figure S3B–E; Figure S4, Supporting Information).

Gene enrichment analysis indicated that metabolic balance in CCA might be altered by CXCL6, especially lipid metabolism (Figure 5A). Lipid metabolism is one of the most prominent metabolic changes in cancer^[^ ^74^ ^]^ and can generate the energy, biofilm components, and signaling molecules required for tumor cell proliferation, invasion, and metastasis while influencing the tumor microenvironment and responses to cancer treatments.^[^ ^75^ ^]^ Lipidomic analysis enabled us to profile CXCL6‐related lipid metabolism changes in CCA and uncovered several core metabolites (Figure 5E–G), in particular the DGs ‘DG 12:0/14:0’, ‘DG (18:0/20:1/0:0)’, and ‘DG (18:0/0:0/18:0)’, which were found at the center of the metabolic network.

The DG metabolite class has a relatively positive function in immune cells in the TIME. In T cells, activation of the T cell receptor (TCR) triggers the production of DGs and stimulates the transcription of genes related to T cell expansion and cytotoxicity.^[^ ^76^, ^77^ ^]^ However, DG enrichment is considered a hallmark of hyporesponsive infiltrating CD8^+^ T cells,^[^ ^78^ ^]^ while genetic ablation of DG kinases (DGKs) enhances the cytotoxic activity of NK cells.^[^ ^58^ ^]^ While targeted inhibitors for DGKα and DGKζ have been developed and investigated preclinically for tumor immunotherapy,^[^ ^79^ ^,^ ^80^ ^]^ the precise functions of DGs in tumor cells remain controversial.^[^ ^81^ ^]^ The downstream pathways and metabolites of DGs are so complex that more research is needed to obtain a proper understanding. In this study, we were unable to clarify the detailed functions of the three core DG metabolites but may analyze this in subsequent studies.

The CXCL6 receptors, CXCR1 and CXCR2, are expressed on tumor and immune cells, and both are important in CXCL6 function.^[^ ^82^ ^]^ We revealed that JAKs could be activated by CXCR1/2 and further phosphorylate STAT3 and PI3K proteins. To our knowledge, this is the first study to directly demonstrate the association between CXCR1/2 and JAKs, which could help clarify the effects of CXCL6 on CCA cells. In addition, CXCR1/2 are important regulator of TANs infiltration.^[^ ^59^ ^]^ Xiong et al. detected CXCL1/2‐induced NETs in esophageal squamous cell carcinoma and found that this augmented NK cells.^[^ ^83^ ^]^ Furthermore, NETs induced by IL‐8 via CXCR1/2 can promote gastric cancer progression.^[^ ^84^ ^]^ Our RNA‐Seq results with cocultured neutrophils strongly suggested that NETs formation is influenced by CXCL6, with the NETs‐associated RAS/MAPK pathway participating in this process as reported.^[^ ^62^, ^63^ ^]^ Thus, we hypothesized that CXCL6 might regulate CCA immunotherapy efficiency and found, using baseline peripheral blood samples, that higher initial CXCL6 levels led to a worse prognosis (Figure 6D–F). Thus, CXCL6 may function as a biomarker for immunotherapy. We also conducted animal experiments in which *CXCL6* silencing in C57BL/6J tumors significantly enhanced the therapeutic efficacy of an anti‐PD‐1 mAb (Figure 6H–J). Changes in the TIME were also detected upon *CXCL6* knockdown. Immunofluorescence assays confirmed that CXCL6 depletion reduced the formation of NETs and increased infiltration of CD8^+^ T cell. A rescue experiment was further conducted to clarify this assumption. As Cl‐amidine inhibited the NETs level caused by CXCL6 overexpression, the infiltration and killing effects of CD8^+^ T cell were also raised, which could help establish the causality between CXCL6 expression, NETs formation, and CD8+ T cell infiltration. This conclusion also mirrors other reports in pancreatic, breast, and bladder cancers, where NETs have been shown to suppress CD8^+^ T cell recruitment to the TIME.^[^ ^70^, ^85^, ^86^ ^]^ Meanwhile, CD8^+^ T cell exhaustion was reported to mediate immunotherapy in CCA,^[^ ^87^ ^]^ as well as other cancers including melanoma, colorectal cancer, and bladder cancer.^[^ ^88^, ^89^, ^90^ ^]^ Hence, we propose that high *CXCL6* expression in CCA reduces CD8^+^ T cell infiltration through NETs formation, eventually causing immunotherapy resistance.

**Figure 8:**
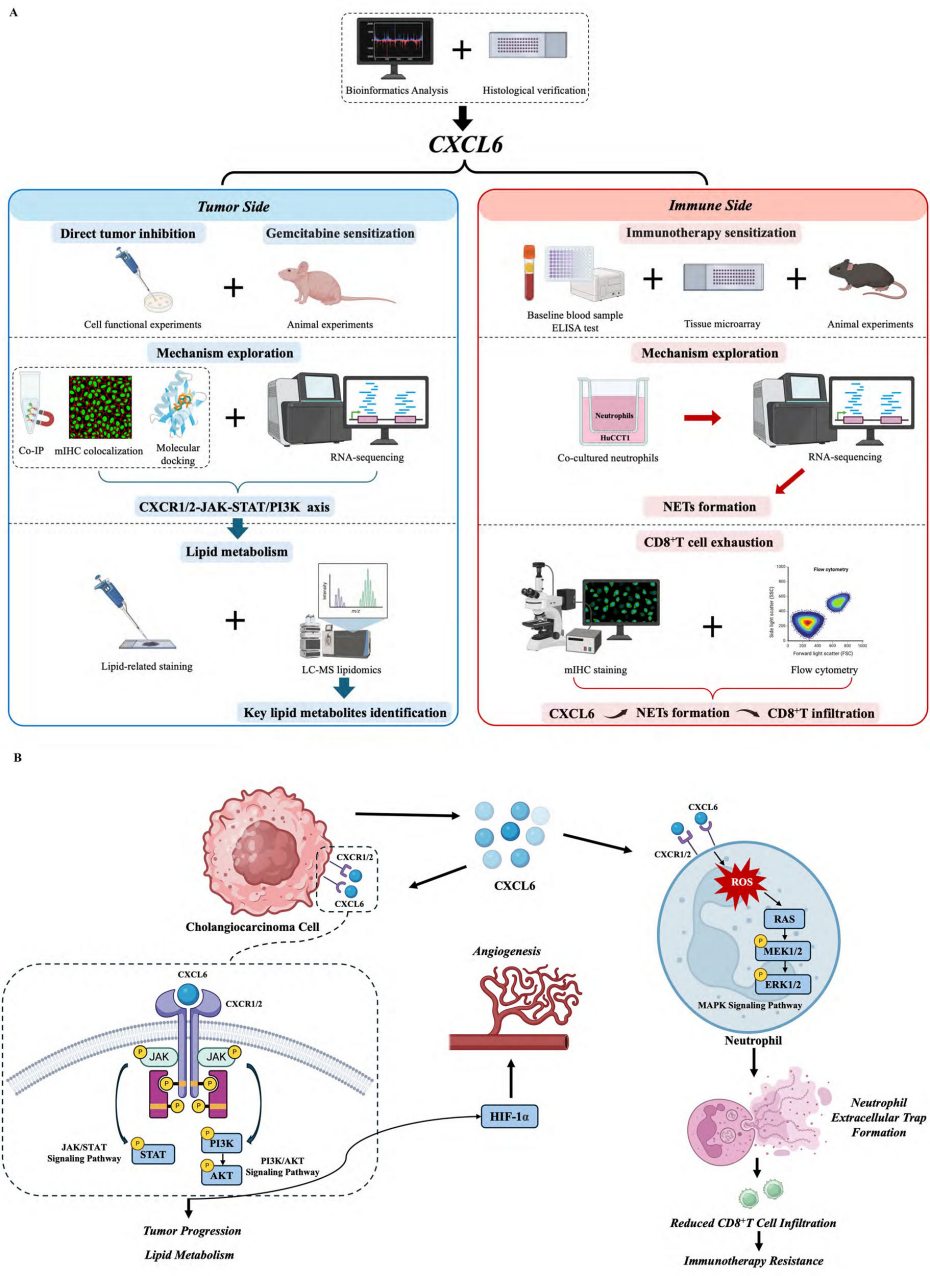
Graphical abstract of the key mechanisms and experimental workflows. A) Experimental workflows of the study. B) Proposed mechanisms of CXCL6 in CCA. Figures were created with BioRender.com.

Through multimodal approaches (**Figure** **8**A), we delineate CXCL6 as a dual driver of CCA progression and immune evasion. In conclusion, the present study has demonstrated CXCL6 as a dual regulator of CCA progression and immunotherapy resistance. Mechanistic investigations showed that CXCL6 activates the CXCR1/2‐JAK‐STAT/PI3K axis in CCA cells through autocrine signaling and reprograms lipid metabolism balance. Concurrently, CXCL6 induces NETs formation via RAS/MAPK signaling, which impedes CD8^+^ T cell infiltration and confers immunotherapy resistance (Figure 8B). Targeting CXCL6 or its downstream effectors may offer a novel sensitizing strategy to augment chemotherapy and immunotherapy responses in CCA patients.

## 4 Experimental Section

### Cell Culture and Extraction

The HuCCT1, RBE, CCLP1, HCCC‐9810, QBC939, and HUVEC cell lines were obtained from the Liver Cancer Institute, Fudan University. The mouse CCA cell line mIC‐23 was established in the Liver Cancer Institute of Fudan University, as reported.^[^ ^65^ ^]^ Human CCA cell lines were cultured in RPMI 1640 medium containing 10% fetal bovine serum (FBS; Gibco, A5670701) and 1% penicillin/streptomycin (Gibco, 15140148) at 37 °C, in an atmosphere containing 5% CO_2_ in an incubator. The mIC‐23 cell line was cultured in DMEM supplemented with 10% FBS (Gibco, A5670701) and 1% penicillin/streptomycin (Gibco, 15140148), with incubation as above. HUVEC cell line was cultured in an endothelial cell medium (SclenCell, 37969), with incubation as above.

Peripheral blood (20 mL per person) was collected from three healthy volunteers into EDTA‐coated tubes. Whole blood was centrifuged at 300 × *g* for 10 min and the middle layer was taken for isolation, then neutrophils were isolated using the MojoSort Whole Blood Human Neutrophil Isolation Kit (BioLegend, 480152). Antibodies and magnetic beads were added to the middle layer in sequence, and each was incubated for more than 20 min. Then the tube was placed in the magnet for 5 min, and the cells poured out were isolated neutrophils. The obtained neutrophils were cultured as for the human CCA cell lines (see above). Where indicated, neutrophils were cocultured with HuCCT1 cells (sh‐NC/sh‐CXCL6 transfected; see below) at a ratio of 10:1.

### Lentivirus, Plasmids, and siRNA Infection

Cells were infected with lentivirus to deliver shRNA‐control (sh‐NC) or shRNA‐CXCL6 (sh‐CXCL6) constructs, using lentiviral preparations purchased from Genomeditech (Shanghai, China). Plasmids for *CXCL6* overexpression and small interfering RNA (siRNA) for *CXCL6* knockdown were sourced from the same supplier. Cells were transfected with plasmids and siRNA using Lipofectamine 3000. The proper infection ratio was tested before the formal experiment and drug screening was conducted after infection. The infection efficiency was confirmed with a Western blot afterward.

### CCA Patients and Tissue Microarray

The “Surgery Cohort” included 192 CCA patients who underwent surgery without pretreatment from January 2018 to December 2020 at the Zhongshan Hospital, Fudan University, Shanghai, China. The “Conversion Therapy Cohort” included 33 CCA patients who underwent surgery after immunotherapy from January 2019 to December 2022 at the same hospital. Written informed consent was obtained from all patients prior to involvement in the study. The study protocol was approved by the Ethics Committee of Zhongshan Hospital (Approval Number B2020‐177R) and was in accordance with the Declaration of Helsinki. Clinicopathological features of patients in “Surgery Cohort” and “Conversion Therapy Cohort” tissue microarray are shown in Tables S1,S2 (Supporting Information). For both cohorts, OS was calculated from the surgery or first cycle of immune‐based therapy to death due to any cause, or censored at the last follow‐up. Disease‐specific survival (DSS) was calculated from the surgery or first cycle of immune‐based therapy to death due to CCA, or censored at the last follow‐up. Event‐free survival (EFS) was calculated from the surgery or first cycle of immune‐based therapy to the first occurrence of any of the following events: disease progression, local or distant recurrence, death due to any cause, or censored at the last follow‐up, etc.

A paraffin‐embedded CCA TMA was constructed and IHC analysis was performed as described below. The antibodies used for IHC staining were: anti‐CXCL6 (Invitrogen, PA5‐115276, 1:100), anti‐CD45 (Servicebio, GB113886‐50), anti‐CD3 (Servicebio, GB13014‐50), anti‐CD8a (Servicebio, GB115692‐50), anti‐CD10 (Servicebio, GB121120‐50, 1:100), anti‐FOXP3 (Servicebio; #GB112325‐50, 1:500), and anti‐CD68 (Servicebio, GB11093‐1‐50, 1:100).

### Animal Studies

Male six‐week‐old C57BL/6J and BALB/c nude mice were purchased from Charles River (Shanghai, China). Animal care and experimental protocols were approved by the Institutional Animal Care and Use Committee (IACUC) of Zhongshan Hospital, Fudan University (20240206‐443), and all mice were fed in a specific pathogen‐free facility. To establish tumors, BALB/c nude mice were injected subcutaneously in the right underarm with 5 × 10^6^ HuCCT1 cells in RPMI 1640 serum‐free medium (200 µL), while C57BL/6J mice were injected in the same manner with 1 × 10^7^ mIC‐23 cells. Two weeks after injection, mice bearing subcutaneous tumors were randomized into groups as indicated below. For tumor‐bearing BALB/c nude mice, treatments were administered intraperitoneally every two days and seven times in total. Tumor‐bearing C57BL/6J mice were treated with 10 mg k g^−1^ anti‐mouse PD‐1 (CD279) (BioXCell, BE0146) or Rat IgG2a isotype control (BioXCell, BE0089) by the same route and with the same frequency.

### Molecular Docking

The 3D structures of JAK1 (PDB6GGH), CXCR1 (PDB2LNL), and CXCR2 (PDB6LFL) proteins were obtained from the RCSB Protein Data Bank (www.rcsb.org/). For docking, structures were converted to PDB files containing all of the polar residues with hydrogens. The GRAMM (https://gramm.compbio.ku.edu/request) semi‐flexible method was adopted for protein–protein docking, and each protein pair was uploaded to a third‐party website to perform the docking, returning the conformation with the lowest binding energy. Models of the resultant protein complexes were analyzed using LigPlus and PyMol.

### HUVEC Tube Formation Assay

Matrigel (60 µL; Corning, 356231) was added to each well of a 24‐well plate and incubated at 37 °C for 10 min. The HUVEC cell line was cultured as mentioned above. Then, HUVEC cells were digested and distributed into the plate at 1 × 10^5^ cells per well. Cells were cultured using a conditioned medium derived from sh‐NC, sh‐CXCL6, and CXCL6‐OE tumor cells. Images were taken 12 h later using a basic bright‐field microscope.

### Flow Cytometry

The identity of isolated neutrophils was confirmed using flow cytometry. Specifically, the collected neutrophils were stained with phycoerythrin (PE) conjugated anti‐human CD45 (BioLegend, 368509) and FITC‐conjugated anti‐human CD16 (BioLegend, 302005) antibodies at 4 °C for 30 min.

In the rescue experiment, mouse tumor tissue was cut into small pieces and placed in appropriate digestive enzymes (TrypLE Express Enzyme, ThermoFisher, 12605036). Tissue was digested in a suitable temperature, dispersed, and filtered into single‐cell suspension. Then single cell suspension was stained by APC‐cy7‐conjungated Zombie (BioLegend, 423106), percpcy5.5‐conjungated CD45 (BioLegend, 103130), APC‐conjungated CD8 (BioLegend, 100712), BV421‐conjungated IFNγ (BioLegend, 505830) and PE‐cy7‐conjungated GzmB (BioLegend, 372214). Flow cytometry was conducted using a BD FACS Aria II flow cytometer and data were analyzed with FlowJo 10.6.2 (FlowJo, LLC).

### Western Blot Assay

Cells and CCA tissue were lysed with RIPA Lysis Buffer (Strong) (Yeasen, 20101ES60). Electrophoresis and membrane transfer were conducted using SDS–PAGE buffer (Servicebio, G2081‐1L) and transfer buffer (Servicebio, G2028‐1L), respectively. Membranes were incubated with primary antibodies at 4 °C overnight, washed three times with TBST (Servicebio, G0004‐500ML) for 10 min, then incubated with secondary antibodies at room temperature for 2 h. Results were detected via the FUSION FX Imaging System (Vilber GmbH, France) using the ultrasensitive ECL FemtoLight Substrate (Epizyme, SQ201). For sh‐NC and sh‐CXCL6 transfected HuCCT1 and RBE cells, three repetitions were performed in each case for western blot.

### Cell Counting Kit‐8 (CCK‐8) Assay

One thousand cells per well were seeded into 96‐well plates. The assay was started by adding 10% CCK‐8 solution (Beyotime, C0037) one day later. After incubation in the dark for 1 h, absorbance measurements were recorded at 450 nm using a SpectraMax iD3/iD5 microplate reader (Molecular Devices, USA). Measurements were repeated every 24 h.

### 5‐Ethynyl‐2′‐Deoxyuridine (EdU) Assay

The BeyoClick EdU Cell Proliferation Kit with Alexa Fluor 488 (Beyotime, C0071S) was used for proliferation assays. Cells in the logarithmic growth phase and in good condition were used for the experiment. Briefly, cells were treated with 10 µm EdU for 2 h, fixed with 4% paraformaldehyde, permeabilized with 0.3% Triton X‐100 for 15 min, then stained using Click Reaction Buffer containing Azide 555 for 30 min followed by Hoechst 33342 for 10 min. Images were acquired using a THUNDER Imaging System (Leica, Germany).

### Colony Formation Assay

Cells were seeded into 6‐well plates (1000 cells per well) and cultured for 15 days. The cell culture medium was changed every three days. For analysis, cells were fixed with 4% paraformaldehyde and stained with Crystal Violet Staining Solution (1.5 mL; Beyotime, C0121‐100 ml) then washed three times with PBS before imaging.

### Wound Healing assay

Cells were seeded into 6‐well plates. Once cultures had reached 95–100% confluency, 200 µL pipette tips were used to introduce wounds. Results were recorded 3 days later using a microscope. Relative migration rates were used to make statistical comparisons.

### Transwell Assay

Cells (2×10^4^ per sample) were seeded into the upper chamber in serum‐free medium and the lower chamber was filled with growth medium containing 20% FBS. After 3 days, cells were fixed and stained with Crystal Violet Staining Solution (Beyotime, C0121‐100 ml). Cells on the lower surface were photographed after removing the redundant (non‐migrated) cells. Migration cell numbers were counted and made statistical comparisons.

### Co‐Immunoprecipitation (Co‐IP) Assay

Cells were lysed with Lysis Buffer (Beyotime, P2179S‐1) and lysates were incubated with anti‐CXCR1 (Absin, 120403), anti‐CXCR2 (Proteintech, 20634‐1‐AP), or anti‐JAK1 (Cell Signaling Technology, 3344T) antibodies at 4 °C overnight. Samples were then incubated with protein A+G magnetic beads (Beyotime, P2179S‐4) at room temperature for 1 h and isolated with a magnet. Immunoprecipitated proteins were detected by western blot.

### Multicolor Immunohistochemistry (mIHC)

Paraffin‐embedded tissue samples were deparaffinized, followed by citrate buffer antigen retrieval at 95 °C and blocking. Samples were then incubated with primary antibody at 4 °C overnight and secondary antibody at 37 °C for 30 min. A DAB Color Development Kit (Servicebio, G1212‐200T) was used for the chromogenic reaction.

### Immunofluorescence

Paraffin‐embedded tissue samples were deparaffinized and blocked with 5% BSA. The samples were subsequently incubated with primary antibody at 4 °C overnight then fluorescent secondary antibody at room temperature for 2 h. Nuclei were stained with DAPI. Images were acquired using a THUNDER Imaging System (Leica, Germany). Statistical analysis was conducted based on ImageJ software.

### Reactive Oxygen Species Detection

Dichlorodihydrofluorescein diacetate (DCFH‐DA) was diluted with serum‐free culture medium to a final concentration of 10 µm. Neutrophils were cultured with HuCCT1 cells as mentioned above. Neutrophils were then collected, resuspended in the diluted DCFH‐DA, and incubated at 37 °C for 20 min. Cells were observed by fluorescence microscopy using 488 nm excitation wavelength and 525 nm emission wavelength.

### RNA Sequencing (RNA‐Seq)

Using the TRIzol reagent (Beyotime, R0016), total RNA was extracted from sh‐NC and sh‐CXCL6 transfected HuCCT1 cells, and from neutrophils after coculture with sh‐WT or sh‐CXCL6 transfected HuCCT1 cells. The concentration and purity of total RNA were checked using a NanoDrop 2000 instrument (Thermo Scientific, MA, USA). Then, RNA‐Seq analysis was performed by LC‐Biotechnology Co., Ltd., Hangzhou, China.

### Gene Ontology (GO) Functional and Kyoto Encyclopedia of Genes and Genomes (KEGG) Pathway Enrichment Analyses

To analyze the possible key pathways, GO functional and KEGG pathway enrichment analyses were conducted and visualized through the “clusterProfler” package based on RNA‐seq data.^[^ ^91^ ^]^ A *p*‐value < 0.05 was considered statistically significant.

### Gene Set Enrichment Analysis (GSEA)

To confirm the enriched KEGG pathways in CCA cells RNA‐seq, GSEA was conducted using the “clusterProfler” package. All genes were ranked based on their log_2_FC, and gene sets with adjusted *p*‐values < 0.05 were considered statistically significant.

### Liquid Chromatography–Mass Spectrometry(LC–MS) Lipidomics

Collected cells were thawed on ice and metabolites were extracted with 80% methanol buffer. All samples were analyzed by LC‐MS. Chromatographic separations were performed using an UltiMate 3000 UPLC System (Thermo Fisher Scientific, Germany) equipped with an ACQUITY UPLC T3 column (100 × 2.1 mm, 1.8 µm; Waters, Milford, CT, USA) for reverse‐phase separation. A TripleTOF 6600 high‐resolution tandem mass spectrometer (SCIEX, Framingham, MA, USA) was used to detect metabolites eluted from the column. After the acquisition, MS data preprocessing was performed using XCMS software, including peak picking, peak grouping, retention time correction, second peak grouping, and annotation of isotopes and adducts. The online KEGG and human metabolome database resources were used to annotate metabolites by matching exact molecular mass data (*m*/*z*) from samples with database values. Statistical analysis was performed in R (version 4.0.0).

### Rescue Experiment

NC/CXCL6‐OE mIC‐23 cells were constructed with plasmids. A total of four groups, “NC+Vector”, “NC+ Cl‐amidine”, “CXCL6‐OE+Vector” and “CXCL6‐OE+Cl‐amidine” were constructed. To establish tumors, C57BL/6J mice were injected subcutaneously in the right underarm with 5 × 10^7^ mIC‐23 cells in RPMI 1640 serum‐free medium (200 µL). Seven days after injection, Cl‐amidine (10 mg k g^−1^)^[^ ^92^ ^]^ or vector was administrated intra‐abdominally daily for NETs formation inhibition, 7 times in total. Acquired tumors were used for flow cytometry tests and mIHC staining.

### Statistical Analysis

Experimental data were expressed as mean ± standard deviation (SD) and analyzed using GraphPad Prism 10.0 (GraphPad Software, San Diego, CA, USA); *P*‐values were calculated via the Student's or Welch's *t‐*test for continuous variables and the chi‐square test for categorical variables. Linear correlations were tested by Pearson correlation analysis. Survival analysis used the Kaplan–Meier method and log‐rank tests. In the figures, the level of statistical significance is indicated as follows: * (*P* < 0.05), ** (*P* < 0.01), or *** (*P* < 0.001).

### Ethical Approval and Consent to Participate

Written informed consent was obtained from all patients before inclusion in the study. The study protocol was approved by the Ethics Committee of Zhongshan Hospital (Approval Number B2020‐177R), and the research was conducted in accordance with the declaration of Helsinki.

Further materials and methods are provided in the supplementary materials and methods.

## Conflict of Interest

The authors declare no conflict of interest.

## Author Contributions

T.H., Z.‐Y.W, B.X. and C.‐J.Z. contributed equally as joint first authors. Conception, design, and development of methodology were done by T.H., Z.‐Y.W., H.‐C.S., and C.H. T.H., Z.‐Y.W., B.X., L.‐N.W., H.‐C.S., C.‐J.Z., Z.‐Y.Y., and S.‐Q.Z. dealt with performing experiments, data curation, and analysis. B.H., X.‐D.Z., Y.‐H.S., J.Z., J.F., H.‐C.S., and C.H. dealt with administration, study supervision, and material support. Writing and visualization were done by T.H. and Z.‐Y.W. Review, editing, and validation were done by B.H., X.‐D.Z., Y.‐H.S., H.‐C.S., and C.H. All authors read and approved the final version of the manuscript.

## Supporting information



Supporting Information

## Data Availability

Research data are not shared.

## References

[1] N. Razumilava , G. J. Gores , Lancet 2014, 383, 2168. 24581682 10.1016/S0140-6736(13)61903-0 PMC4069226

[2] A. Rizzo , G. Brandi , Expert Rev. Gastroenterol. Hepatol. 2021, 15, 483. 33307876 10.1080/17474124.2021.1864325

[3] J. W. Valle , R. K. Kelley , B. Nervi , D. Y. Oh , A. X. Zhu , Lancet 2021, 397, 428. 33516341 10.1016/S0140-6736(21)00153-7

[4] A. B. Benson , M. I. D'Angelica , D. E. Abbott , T. A. Abrams , S. R. Alberts , D. A. Anaya , R. Anders , C. Are , D. Brown , D. T. Chang , J. Cloyd , A. M. Covey , W. Hawkins , R. Iyer , R. Jacob , A. Karachristos , R. K. Kelley , R. Kim , M. Palta , J. O. Park , V. Sahai , T. Schefter , J. K. Sicklick , G. Singh , D. Sohal , S. Stein , G. G. Tian , J.‐N. Vauthey , A. P. Venook , L. J. Hammond , et al., J. Natl. Compr. Cancer Network 2019, 17, 302. 10.6004/jnccn.2019.0019 30959462

[5] C. Morizane , T. Okusaka , J. Mizusawa , H. Katayama , M. Ueno , M. Ikeda , M. Ozaka , N. Okano , K. Sugimori , A. Fukutomi , H. Hara , N. Mizuno , H. Yanagimoto , K. Wada , K. Tobimatsu , K. Yane , S. Nakamori , H. Yamaguchi , A. Asagi , S. Yukisawa , Y. Kojima , K. Kawabe , Y. Kawamoto , R. Sugimoto , T. Iwai , K. Nakamura , H. Miyakawa , T. Yamashita , A. Hosokawa , T. Ioka , et al., Ann. Oncol. 2019, 30, 1950. 31566666 10.1093/annonc/mdz402

[6] J. M. Phelip , J. Desrame , J. Edeline , E. Barbier , E. Terrebonne , P. Michel , H. Perrier , L. Dahan , V. Bourgeois , F. K. Akouz , E. Soularue , V. L. Ly , Y. Molin , T. Lecomte , F. Ghiringhelli , R. Coriat , S. Louafi , C. Neuzillet , S. Manfredi , D. Malka , J. Clin. Oncol. 2022, 40, 262. 34662180 10.1200/JCO.21.00679

[7] E. Woods , D. Le , B. K. Jakka , A. Manne , Cancers 2022, 14, 2137. 35565266 10.3390/cancers14092137 PMC9105885

[8] J. N. Primrose , R. P. Fox , D. H. Palmer , H. Z. Malik , R. Prasad , D. Mirza , A. Anthony , P. Corrie , S. Falk , M. Finch‐Jones , H. Wasan , P. Ross , L. Wall , J. Wadsley , J. T. R. Evans , D. Stocken , R. Praseedom , Y. T. Ma , B. Davidson , J. P. Neoptolemos , T. Iveson , J. Raftery , S. Zhu , D. Cunningham , O. J. Garden , C. Stubbs , J. W. Valle , J. Bridgewater , J. Primrose , R. Fox , et al., Lancet Oncol. 2019, 20, 2137663.

[9] H. A. Burris , T. Okusaka , A. Vogel , M. A. Lee , H. Takahashi , V. Breder , J.‐F. Blanc , J. Li , M. Bachini , M. Zotkiewicz , J. Abraham , N. Patel , J. Wang , M. Ali , N. Rokutanda , G. Cohen , D.‐Y. Oh , Lancet Oncol. 2024, 25, 626. 38697156 10.1016/S1470-2045(24)00082-2

[10] M. Z. Jin , W. L. Jin , Signal Transduction Targeted Ther. 2020, 5, 166. 10.1038/s41392-020-00280-x PMC7447642 32843638

[11] L. Fabris , J. B. Andersen , L. Fouassier , J. Hepatol. 2020, 73, 1007. 32900521 10.1016/j.jhep.2020.07.017

[12] D. Leyva‐Illades , M. McMillin , M. Quinn , S. Demorrow , Transl. Gastrointest. Cancer 2012, 1, 71. 23002431 PMC3448449

[13] Z. Y. Mao , G. Q. Zhu , M. Xiong , L. Ren , L. Bai , World J. Gastroenterol. 2015, 21, 4961. 25945010 10.3748/wjg.v21.i16.4961 PMC4408469

[14] F.‐M. Gu , Q. Gao , G.‐M. Shi , X. Zhang , J. Wang , J.‐H. Jiang , X.‐Y. Wang , Y.‐H. Shi , Z.‐B. Ding , J. Fan , J. Zhou , Ann. Surg. Oncol. 2012, 19, 2506. 22411204 10.1245/s10434-012-2268-8

[15] J. M. Adrover , S. A. C. McDowell , X. Y. He , D. F. Quail , M. Egeblad , Cancer Cell 2023, 41, 505. 36827980 10.1016/j.ccell.2023.02.001 PMC10280682

[16] S. Raza , S. Rajak , A. Tewari , P. Gupta , N. Chattopadhyay , R. A. Sinha , B. Chakravarti , Semin. Cancer Biol. 2022, 86, 1105. 10.1016/j.semcancer.2021.12.011 PMC7613720 34979274

[17] H. Li , M. Wu , X. Zhao , MedComm 2020, 3, 147. 10.1002/mco2.147 PMC9175564 35702353

[18] J. Lu , W. Xu , J. Qian , S. Wang , B. Zhang , L. Zhang , R. Qiao , M. Hu , Y. Zhao , X. Zhao , B. Han , BMC Med. Genomics 2019, 12, 38. 30871526 10.1186/s12920-019-0482-y PMC6416828

[19] N. Unver , Med. Oncol. 2021, 38, 68. 33983509 10.1007/s12032-021-01517-7

[20] T. Wu , W. Yang , A. Sun , Z. Wei , Q. Lin , Cancers 2022, 15, 167. 36612163 10.3390/cancers15010167 PMC9818145

[21] L. Li , Y. Xia , X. Ji , H. Wang , Z. Zhang , P. Lu , Q. Ding , D. Wang , M. Liu , Exp. Cell Res. 2021, 407, 167112801. 10.1016/j.yexcr.2021.112801 34461107

[22] J. Bian , J. Fu , X. Wang , J. Lee , G. Brar , F. E. Escorcia , M. Cam , C. Xie , Stem Cells Int. 2022, 2022, 13558200. 10.1155/2022/3558200 PMC9076354 35530414

[23] M. Song , J. He , Q.‐Z. Pan , J. Yang , J. Zhao , Y.‐J. Zhang , Y. Huang , Y. Tang , Q. Wang , J. He , J. Gu , Y. Li , S. Chen , J. Zeng , Z.‐Q. Zhou , C. Yang , Y. Han , H. Chen , T. Xiang , D.‐S. Weng , J.‐C. Xia , Hepatology 2021, 73, 1717. 33682185 10.1002/hep.31792

[24] J. Li , Z. Tang , H. Wang , W. Wu , F. Zhou , H. Ke , W. Lu , S. Zhang , Y. Zhang , S. Yang , S. Ni , J. Huang , Biomed. Pharmacother. 2018, 97, 1182. 29136957 10.1016/j.biopha.2017.11.004

[25] Z.‐G. Shan , J. Chen , J.‐S. Liu , J.‐Y. Zhang , T.‐T. Wang , Y.‐S. Teng , F.‐Y. Mao , P. Cheng , Q.‐M. Zou , W.‐Y. Zhou , L.‐S. Peng , Y.‐L. Zhao , Y. Zhuang , Clin. Transl. Med. 2021, 11, 484. 10.1002/ctm2.484 PMC8236123 34185422

[26] S. S. Glaser , E. Gaudio , G. Alpini , Curr. Opin. Gastroenterol. 2010, 26, 246. 20061944 10.1097/MOG.0b013e3283369d19 PMC2893138

[27] R. K. Jain , Science 2005, 307, 58.15637262

[28] J. J. Harding , D. N. Khalil , L. Fabris , G. K. Abou‐Alfa , J. Hepatol. 2023, 78, 217. 36150578 10.1016/j.jhep.2022.09.004 PMC11111174

[29] M. J. Williams , X. Wang , H. P. Bastos , G. Grondys‐Kotarba , Q. Wu , S. Jin , C. S. Johnson , N. Mende , E. F. Calderbank , M. Wantoch , H. J. Park , G. Mantica , R. L. Hannah , N. K. Wilson , D. C. Pask , T. L. Hamilton , S. J. Kinstone , R. Asby , R. Sneade , E. J. Baxtor , P. J. Campbell , G. S. Vassiliou , E. Laurenti , J. Li , B. Gottgens , A. R. Green , Blood Adv. 2025, 9, 291. 39374575 10.1182/bloodadvances.2024014046 PMC7617191

[30] B. Gao , X. Shen , G. Kunos , Q. Meng , I. D. Goldberg , E. M. Rosen , S. Fan , FEBS Lett. 2001, 488, 179. 11163768 10.1016/s0014-5793(00)02430-3

[31] R. Garcia , T. L. Bowman , G. Niu , H. Yu , S. Minton , C. A. Muro‐Cacho , C. E. Cox , R. Falcone , R. Fairclough , S. Parsons , A. Laudano , A. Gazit , A. Levitzki , A. Kraker , R. Jove , Oncogene 2001, 20, 2499. 11420660 10.1038/sj.onc.1204349

[32] O. V. Smirnova , T. Y. Ostroukhova , R. L. Bogorad , World J. Gastroenterol. 2007, 13, 6478. 18161917 10.3748/wjg.v13.i48.6478 PMC4611286

[33] F. Z. Mokhfi , M. Al Amin , M. Zehravi , S. H. Sweilam , U. V. N. V. Arjun , J. K. Gupta , B. Vallamkonda , A. Balakrishnan , M. Challa , J. Singh , P. D. Prasad , S. S. Ali , I. Ahmad , K. Doukani , T. B. Emran , Chem.‐Biol. Interact. 2024, 402, 111218. 39209016 10.1016/j.cbi.2024.111218

[34] M. Laplante , D. M. Sabatini , Cell 2012, 149, 274. 22500797 10.1016/j.cell.2012.03.017 PMC3331679

[35] X. Wang , Y. Dai , X. Zhang , K. Pan , Y. Deng , J. Wang , T. Xu , Cancer Biol. Ther. 2021, 22, 30. 33241954 10.1080/15384047.2020.1842705 PMC7834049

[36] S. Sitaru , A. Budke , R. Bertini , M. Sperandio , Intern. Emerg. Med. 2023, 18, 1647. 37249756 10.1007/s11739-023-03309-5 PMC10227827

[37] A. Mantovani , M. A. Cassatella , C. Costantini , S. Jaillon , Nat. Rev. Immunol. 2011, 11, 519. 21785456 10.1038/nri3024

[38] Z. Zhou , G. Xia , Z. Xiang , M. Liu , Z. Wei , J. Yan , W. Chen , J. Zhu , N. Awasthi , X. Sun , K.‐M. Fung , Y. He , M. Li , C. Zhang , Clin. Cancer Res. 2019, 25, 3317. 30796034 10.1158/1078-0432.CCR-18-3567 PMC8955044

[39] T. M. Cunha , M. M. Barsante , A. T. Guerrero , W. A. Verri , S. H. Ferreira , F. M. Coelho , R. Bertini , C. Di Giacinto , M. Allegretti , F. Q. Cunha , M. M. Teixeira , Br. J. Pharmacol. 2008, 154, 460. 18362895 10.1038/bjp.2008.94 PMC2442455

[40] C. R. Glassman , N. Tsutsumi , R. A. Saxton , P. J. Lupardus , K. M. Jude , K. C. Garcia , Science 2022, 376, 163. 35271300 10.1126/science.abn8933 PMC9306331

[41] J. J. O'Shea , D. M. Schwartz , A. V. Villarino , M. Gadina , I. B. McInnes , A. Laurence , Annu. Rev. Med. 2015, 66, 311. 25587654 10.1146/annurev-med-051113-024537 PMC5634336

[42] X. Hu , J. Li , M. Fu , X. Zhao , W. Wang , Signal Transduction Targeted Ther. 2021, 6, 402. 10.1038/s41392-021-00791-1 PMC8617206 34824210

[43] A. Singh , M. M. Copeland , P. J. Kundrotas , I. A. Vakser , Methods Mol. Biol. 2024, 2714, 101. 37676594 10.1007/978-1-0716-3441-7_5

[44] J. R. White , J. M. Lee , P. R. Young , R. P. Hertzberg , A. J. Jurewicz , M. A. Chaikin , K. Widdowson , J. J. Foley , L. D. Martin , D. E. Griswold , H. M. Sarau , J. Biol. Chem. 1998, 273, 10095. 9553055 10.1074/jbc.273.17.10095

[45] R. Bertini , M. Allegretti , C. Bizzarri , A. Moriconi , M. Locati , G. Zampella , M. N. Cervellera , V. Di Cioccio , M. C. Cesta , E. Galliera , F. O. Martinez , R. Di Bitondo , G. Troiani , V. Sabbatini , G. D'Anniballe , R. Anacardio , J. C. Cutrin , B. Cavalieri , F. Mainiero , R. Strippoli , P. Villa , M. Di Girolamo , F. Martin , M. Gentile , A. Santoni , D. Corda , G. Poli , A. Mantovani , P. Ghezzi , F. Colotta , Proc. Natl. Acad. Sci. 2004, 101, 11791. 15282370 10.1073/pnas.0402090101 PMC511013

[46] M. Kilic‐Eren , T. Boylu , V. Tabor , Cancer Cell Int. 2013, 13, 36. 23590596 10.1186/1475-2867-13-36 PMC3637483

[47] F. F. Tam , K. L. Ning , M. Lee , J. M. Dumlao , J. C. Choy , Mol. Immunol. 2023, 160, 12. 37295053 10.1016/j.molimm.2023.06.001

[48] Y. J. Li , C. Zhang , A. Martincuks , A. Herrmann , H. Yu , Nat. Rev. Cancer 2023, 23, 115. 36596870 10.1038/s41568-022-00537-3

[49] F. Fontana , G. Giannitti , S. Marchesi , P. Limonta , Int. J. Biol. Sci. 2024, 20, 3113. 38904014 10.7150/ijbs.89942 PMC11186371

[50] T. Li , J. Weng , Y. Zhang , K. Liang , G. Fu , Y. Li , X. Bai , Y. Gao , Cell Death Dis. 2019, 10, 619. 31409773 10.1038/s41419-019-1828-2 PMC6692326

[51] R.‐H. Tu , S.‐Z. Wu , Z.‐N. Huang , Q. Zhong , Y.‐H. Ye , C.‐H. Zheng , J.‐W. Xie , J.‐B. Wang , J.‐X. Lin , Q.‐Y. Chen , C.‐M. Huang , M. Lin , J. Lu , L.‐L. Cao , P. Li , Cancer Res. 2023, 83, 3868. 38037454 10.1158/0008-5472.CAN-23-1012

[52] Q. Ma , H. Jiang , L. Ma , G. Zhao , Q. Xu , D. Guo , N. He , H. Liu , Z. Meng , J. Liu , L. Zhu , Q. Lin , X. Wu , M. Li , S. Luo , J. Fang , Z. Lu , Proc. Natl. Acad. Sci. U. S. A. 2023, 120, 2209435120. 10.1073/pnas.2209435120 PMC10104498 37011206

[53] Y. Ping , J. Shan , H. Qin , F. Li , J. Qu , R. Guo , D. Han , W. Jing , Y. Liu , J. Liu , Z. Liu , J. Li , D. Yue , F. Wang , L. Wang , B. Zhang , B. Huang , Y. Zhang , Immunity 2024, 57, 21222122. 10.1016/j.immuni.2024.08.003 39208806

[54] A. Mukherjee , D. Bezwada , F. Greco , M. Zandbergen , T. Shen , C.‐Y. Chiang , M. Tasdemir , J. Fahrmann , D. Grapov , M. R. La Frano , H. S. Vu , B. Faubert , J. W. Newman , L. A. McDonnell , L. Nezi , O. Fiehn , R. J. DeBerardinis , E. Lengyel , Nat. Metab. 2023, 5, 1563. 37653041 10.1038/s42255-023-00879-8 PMC12175003

[55] C. B. Nava Lauson , S. Tiberti , P. A. Corsetto , F. Conte , P. Tyagi , M. Machwirth , S. Ebert , A. Loffreda , L. Scheller , D. Sheta , Z. Mokhtari , T. Peters , A. T. Raman , F. Greco , A. M. Rizzo , A. Beilhack , G. Signore , N. Tumino , P. Vacca , L. A. McDonnell , A. Raimondi , P. D. Greenberg , J. B. Huppa , S. Cardaci , I. Caruana , S. Rodighiero , L. Nezi , T. Manzo , Cell Metab. 2023, 35, 633. 36898381 10.1016/j.cmet.2023.02.013

[56] L. Sun , C. Suo , T. Zhang , S. Shen , X. Gu , S. Qiu , P. Zhang , H. Wei , W. Ma , R. Yan , R. Chen , W. Jia , J. Cao , H. Zhang , P. Gao , Nat. Chem. Biol. 2023, 19, 1492. 37500770 10.1038/s41589-023-01391-6

[57] P. U. Prinz , A. N. Mendler , I. Masouris , L. Durner , R. Oberneder , E. Noessner , J. Immunol. 2012, 188, 5990. 22573804 10.4049/jimmunol.1103028

[58] E. Yang , B. K. Singh , A. M. Paustian , T. Kambayashi , J. Immunol. 2016, 197, 934. 27342844 10.4049/jimmunol.1600581 PMC4935923

[59] C. Alfaro , A. Teijeira , C. Oñate , G. Pérez , M. F. Sanmamed , M. P. Andueza , D. Alignani , S. Labiano , A. Azpilikueta , A. Rodriguez‐Paulete , S. Garasa , J. P. Fusco , A. Aznar , S. Inogés , M. De Pizzol , M. Allegretti , J. Medina‐Echeverz , P. Berraondo , J. L. Perez‐Gracia , I. Melero , Clin. Cancer Res. 2016, 22, 3924. 26957562 10.1158/1078-0432.CCR-15-2463

[60] C. Schauer , C. Janko , L. E. Munoz , Y. Zhao , D. Kienhöfer , B. Frey , M. Lell , B. Manger , J. Rech , E. Naschberger , R. Holmdahl , V. Krenn , T. Harrer , I. Jeremic , R. Bilyy , G. Schett , M. Hoffmann , M. Herrmann , Nat. Med. 2014, 20, 511. 24784231 10.1038/nm.3547

[61] Y. Kuang , U. Parthasarathy , R. Martinelli , STAR Protoc. 2023, 4, 102497. 37590147 10.1016/j.xpro.2023.102497 PMC10461015

[62] A. Hakkim , T. A. Fuchs , N. E. Martinez , S. Hess , H. Prinz , A. Zychlinsky , H. Waldmann , Nat. Chem. Biol. 2011, 7, 75. 21170021 10.1038/nchembio.496

[63] T. DeSouza‐Vieira , A. Guimarães‐Costa , N. C. Rochael , M. N. Lira , M. T. Nascimento , P. D. S. Lima‐Gomez , R. M. Mariante , P. M. Persechini , E. M. Saraiva , J. Leukocyte Biol. 2016, 100, 801. 27154356 10.1189/jlb.4A0615-261RR PMC5014744

[64] L. Vitkov , J. Krunic , J. Dudek , M. R. Bobbili , J. Grillari , B. Hausegger , I. Mladenovic , N. Stojanovic , W. D. Krautgartner , H. Oberthaler , C. Schauer , M. Herrmann , J. Singh , B. Minnich , M. Hannig , Int. J. Mol. Sci. 2024, 25, 3314. 38542287 10.3390/ijms25063314 PMC10970663

[65] T. N. Mayadas , X. Cullere , C. A. Lowell , Annu. Rev. Pathol. 2014, 9, 181. 24050624 10.1146/annurev-pathol-020712-164023 PMC4277181

[66] B.‐Y. Sun , Z.‐T. Wang , K.‐Z. Chen , Y. Song , J.‐F. Wu , D. Zhang , G.‐Q. Sun , J. Zhou , J. Fan , B. Hu , Y. Yi , S.‐J. Qiu , Cell Death Discovery 2024, 10, 304. 38926350 10.1038/s41420-024-02079-z PMC11208581

[67] J.‐C. Lu , L.‐L. Wu , Y.‐N. Sun , X.‐Y. Huang , C. Gao , X.‐J. Guo , H.‐Y. Zeng , X.‐D. Qu , Y. Chen , D. Wu , Y.‐Z. Pei , X.‐L. Meng , Y.‐M. Zheng , C. Liang , P.‐F. Zhang , J.‐B. Cai , Z.‐B. Ding , G.‐H. Yang , N. Ren , C. Huang , X.‐Y. Wang , Q. Gao , Q.‐M. Sun , Y.‐H. Shi , S.‐J. Qiu , A.‐W. Ke , G.‐M. Shi , J. Zhou , Y.‐D. Sun , J. Fan , Nat. Commun. 2024, 15, 621. 38245530 10.1038/s41467-024-44795-1 PMC10799889

[68] J. S. Knight , W. Luo , A. A. O'Dell , S. Yalavarthi , W. Zhao , V. Subramanian , C. Guo , R. C. Grenn , P. R. Thompson , D. T. Eitzman , M. J. Kaplan , Circ. Res. 2014, 114, 947. 24425713 10.1161/CIRCRESAHA.114.303312 PMC4185401

[69] F. C. Onyeogaziri , R. Smith , M. Arce , H. Huang , I. Erzar , C. Rorsman , M. Malinverno , F. Orsenigo , V. Sundell , D. Fernando , G. Daniel , M. Niemelä , A. Laakso , B. R. Jahromi , A.‐K. Olsson , P. U. Magnusson , Nat. Cardiovasc. Res. 2024, 3, 1549. 39632986 10.1038/s44161-024-00577-y PMC11634782

[70] S. Shinde‐Jadhav , J. J. Mansure , R. F. Rayes , G. Marcq , M. Ayoub , R. Skowronski , R. Kool , F. Bourdeau , F. Brimo , J. Spicer , W. Kassouf , Nat. Commun. 2021, 12, 2776. 33986291 10.1038/s41467-021-23086-z PMC8119713

[71] C. Zhou , Y. Gao , P. Ding , T. Wu , G. Ji , Cell Death Discovery 2023, 9, 212. 37393391 10.1038/s41420-023-01524-9 PMC10314943

[72] L. Wu , J. Yan , Y. Bai , F. Chen , X. Zou , J. Xu , A. Huang , L. Hou , Y. Zhong , Z. Jing , Q. Yu , X. Zhou , Z. Jiang , C. Wang , M. Cheng , Y. Ji , Y. Hou , R. Luo , Q. Li , L. Wu , J. Cheng , P. Wang , D. Guo , W. Huang , J. Lei , S. Liu , Y. Yan , Y. Chen , S. Liao , Y. Li , et al., Cell Res. 2023, 33, 585. 37337030 10.1038/s41422-023-00831-1 PMC10397313

[73] M. Li , J. Song , P. Pytel , Folia Neuropathol. 2014, 52, 234. 25310734 10.5114/fn.2014.45564

[74] G. Pascual , S. A. Benitah , Front. Oncol. 2024, 14, 1435480. 39391242 10.3389/fonc.2024.1435480 PMC11464260

[75] X. Bian , R. Liu , Y. Meng , D. Xing , D. Xu , Z. Lu , J. Exp. Med. 2021, 218, 20201606. 10.1084/jem.20201606 PMC7754673 33601415

[76] M. A. Sanjua?n , B. R. R. Pradet‐Balade , D. R. Jones , C. Marti?nez‐A , J. C. Stone , J. A. Garcia‐Sanz , I. Me?rida , J. Immunol. 2003, 170, 2877. 12626538 10.4049/jimmunol.170.6.2877

[77] S. Krishna , X. Zhong , Crit. Rev. Immunol. 2013, 33, 97. 23582058 10.1615/critrevimmunol.2013006696 PMC3689416

[78] E. M. Wesley , G. Xin , D. McAllister , S. Malarkannan , D. K. Newman , M. B. Dwinell , W. Cui , B. D. Johnson , M. J. Riese , Immunohorizons 2018, 2, 107. 30027154 10.4049/immunohorizons.1700055 PMC6048965

[79] Y. Jiang , F. Sakane , H. Kanoh , J. P. Walsh , Biochem. Pharmacol. 2000, 59, 763. 10718334 10.1016/s0006-2952(99)00395-0

[80] R. Offringa , C. Olesch , F. Cichon , M. Grees , N. Schmees , U. Roehn , J. Immunother. Cancer 2023, 11, A1029.

[81] M. Cooke , M. G. Kazanietz , Sci. Signaling 2022, 15, abo0264. 10.1126/scisignal.abo0264 PMC12985357 35412850

[82] H. Bahudhanapati , J. Tan , R. M. Apel , B. Seeliger , J. Schupp , X. Li , D. I. Sullivan , J. Sembrat , M. Rojas , T. Tabib , E. Valenzi , R. Lafyatis , N. Mitash , R. Hernandez Pineda , C. Jawale , D. Peroumal , P. Biswas , J. Tedrow , T. Adams , N. Kaminski , W. A. Wuyts , J. F. McDyer , K. F. Gibson , J. K. Alder , M. Königshoff , Y. Zhang , M. Nouraie , A. Prasse , D. J. Kass , Eur. Respir. J. 2024, 63, 2300088. 37918852 10.1183/13993003.00088-2023

[83] G. Xiong , Z. Chen , Q. Liu , F. Peng , C. Zhang , M. Cheng , R. Ling , S. Chen , Y. Liang , D. Chen , Q. Zhou , J. Immunother. Cancer 2024, 12, 008662. 10.1136/jitc-2023-008662 PMC11086492 38724465

[84] Q. Wang , Y. Zhang , W. Ding , C. Feng , Y. Wang , X. Wei , Z. Qu , H. Wang , X. Liu , H. Wang , K. Gu , Genes Dis. 2024, 11, 575. 37692473 10.1016/j.gendis.2023.03.025 PMC10491907

[85] S. B. Coffelt , K. Kersten , C. W. Doornebal , J. Weiden , K. Vrijland , C.‐S. Hau , N. J. M. Verstegen , M. Ciampricotti , L. J. A. C. Hawinkels , J. Jonkers , K. E. de Visser , Nature 2015, 522, 345. 25822788 10.1038/nature14282 PMC4475637

[86] Y. Zhang , V. Chandra , E. Riquelme Sanchez , P. Dutta , P. R. Quesada , A. Rakoski , M. Zoltan , N. Arora , S. Baydogan , W. Horne , J. Burks , H. Xu , P. Hussain , H. Wang , S. Gupta , A. Maitra , J. M. Bailey , S. J. Moghaddam , S. Banerjee , I. Sahin , P. Bhattacharya , F. McAllister , J. Exp. Med. 2020, 217, 20190354. 10.1084/jem.20190354 PMC7953739 32860704

[87] C. Chen , F. Zhao , J. Peng , D. Zhao , L. Xu , H. Li , S. Ma , X. Peng , X. Sheng , Y. Sun , T. Wang , H. Dong , Y. Ding , Z. Wu , X. Liang , L. Gao , H. Wang , C. Ma , C. Li , Cell Rep. Med. 2024, 5, 101686. 39168104 10.1016/j.xcrm.2024.101686 PMC11384939

[88] H. Tao , C. Jin , L. Zhou , Z. Deng , X. Li , W. Dang , S. Fan , B. Li , F. Ye , J. Lu , X. Kong , C. Liu , C. Luo , Y. Zhang , Cancer Res. 2024, 84, 419. 37991725 10.1158/0008-5472.CAN-23-1082

[89] F. He , Z. Wu , C. Liu , Y. Zhu , Y. Zhou , E. Tian , R. Rosin‐Arbesfeld , D. Yang , M.‐W. Wang , D. Zhu , Signal Transduction Targeted Ther. 2024, 9, 139. 10.1038/s41392-024-01838-9 PMC11137111 38811552

[90] A. Yu , J. Hu , L. Fu , G. Huang , D. Deng , M. Zhang , Y. Wang , G. Shu , L. Jing , H. Li , X. Chen , T. Yang , J. Wei , Z. Chen , X. Zu , J. Luo , J. Immunother. Cancer 2023, 11, 007230. 10.1136/jitc-2023-007230 PMC10565151 37802603

[91] T. Wu , E. Hu , S. Xu , M. Chen , P. Guo , Z. Dai , T. Feng , L. Zhou , W. Tang , L. Zhan , X. Fu , S. Liu , X. Bo , G. Yu , Innovation 2021, 2, 100141. 34557778 10.1016/j.xinn.2021.100141 PMC8454663

[92] J. S. Knight , V. Subramanian , A. A. O'Dell , S. Yalavarthi , W. Zhao , C. K. Smith , J. B. Hodgin , P. R. Thompson , M. J. Kaplan , Ann. Rheum. Dis. 2015, 74, 2199. 25104775 10.1136/annrheumdis-2014-205365 PMC4320672

