# Supplementary material for: CXCL6 Reshapes Lipid Metabolism and Induces Neutrophil Extracellular Trap Formation in Cholangiocarcinoma Progression and Immunotherapy Resistance
Source: Adv Sci (Weinh). 2025 Apr 30;12(27):2503009. doi: 10.1002/advs.202503009 (PMC12279165; doi:10.1002/advs.202503009)
Supplement: Supplementary file 1 — Supporting Information [file ADVS-12-2503009-s001.pdf]

ADVANCED SCIENCE
Open Access

Supporting Information

for *Adv. Sci.*, DOI 10.1002/advs.202503009

CXCL6 Reshapes Lipid Metabolism and Induces Neutrophil Extracellular Trap Formation in Cholangiocarcinoma Progression and Immunotherapy Resistance

Tian He, Zi-Yi Wang, Bin Xu, Cheng-Jie Zhong, Lu-Na Wang, Huan-Chen Shi, Zi-Yue Yang, Shi-Qi Zhou, Hui Li, Bo Hu, Xiao-Dong Zhu, Ying-Hao Shen, Jian Zhou, Jia Fan, Hui-Chuan Sun and Cheng Huang**

Supplementary information

1. Primers used in RT-qPCR analysis

CXCL6 forward: CCACTATGAGCCTCCCGTCCAG
CXCL6 reverse: AGCACAGCAGAGACAGGACCAG

2. Sequences of shRNA, plasmids and siRNAs

Plasmid：
PGMLV-CMV-H_CXCL6-3×Flag-EF1-ZsGreen1-T2A-Puro(for human)：
GCCACCATGAGCCTCCCGTCCAGCCGCGCGGCCCGTGTCCCGGGTCCTTCGGGCTCCT
TGTGCGCGCTGCTCGCGCTGCTGCTCCTGCTGACGCCGCCGGGGCCCCTCGCCAGCGC
TGGTCCTGTCTCTGCTGTGCTGACAGAGCTGCGTTGCACTTGTTTACGCGTTACGCTGA
GAGTAAACCCCAAAACGATTGGTAAACTGCAGGTGTTCCCCGCAGGCCCGCAGTGCTC
CAAGGTGGAAGTGGTAGCCTCCCTGAAGAACGGGAAGCAAGTTTGTCTGGACCCGGA
AGCCCCTTTTCTAAAGAAAGTCATCCAGAAAATTTTGGACAGTGGAAACAAGAAAAA
C

pcDNA3.1(-)-EGFP-CXCL6(for mice):
ATGGTGAGCAAGGGCGAGGAGCTGTTTACCGGGGTGGTGCCCATCCTGGTCGAGCTG
GACGGCGACGTAAACGGCCACAAGTTCAGCGTGTCCGGCGAGGGCGAGGGCGATGCC
ACCTACGGCAAGCTGACCCTGAAGTTCATCTGCACCACCGGCAAACTGCCCGTGCCCT
GGCCCACCCTCGTGACCACCCTGACCTACGGCGTGCAGTGCTTCAGCCGCTACCCCGA
CCACATGAAGCAGCACGACTTCTTCAAGTCCGCCATGCCCGAGGGCTACGTCCAGGAG
CGCACCATCTTCTTCAAGGACGACGGCAACTACAAGACCCGCGCCGAGGTGAAGTT
CGAGGGCGACACCCTGGTGAACCGCATCGAGCTGAAGGGCATCGACTTCAAGGAGGA
CGGCAAACATCCTGGGGCACAAGCTGGAGTACAACTACAACAGCCACAACGTCTATAT
CATGGCCGACAAGCAGAAGAACGGCATCAAGGTGAACTTCAAGATCCGCCACAACAT
CGAGGACGGCAGCGTGCAGCTCGCCGACCACTACCAGCAGAACACCCCCATCGGCCG
ACGGCCCCGTGCTGCTGCCCGACAACCACTACCTGAGCACCCAGTCCGCCCTGAGCA
AAGACCCCAACGAGAAGCGCGATCACATGGTCCTGCTGGAGTTCGTGACCGCCGCCG
GGATCACTCTCGGCCATGGACGAGCTGTACAAGATGAGCCTCCAGCTCCGCAGCTCCG
CCCGCCATCCCAGCGGTTCCATCTCGCCATTCATGCGGATGGCCCCGCTGGCCATTTCT
GTTGCTGTTTACGCTGCCGCAGCATCTAGCTGAAGCTGCCCCTTCCCTCAGTCATAGCC
GCAACGGAGCTGCGTTGTGTTTGCTTAACCGTAACTCCAAAAATTAATCCCAAATTGAT
CGCTAATTTGGAGGTGATCCCTGCAGGTCCACAGTGCCCTACGGTGGAAGTCATAGCT
AAACTGAAAAACCAGAAGGAGGTCTGTCTGGATCCAGAAGCTCCTGTGATAAAGAAA
ATCATTCTGAAAATATTGGGCAGTGACAAAAAGAAAGCTAAGCGGCCATGCACTCGCA
GTGGAAAGAACGGCGAGTGTTCAATAG

shRNA
H_CXCL6-shRNA1 (PGMLV-Puro)：
GGACTATCATATGCTTACCGTAACTTGAAAGTATTTCGATTTCTTGGCTTTATATATCTTG

TGGAAAGGACGAGGATCCGCGCGTTACGCTGAGAGTAAACCTCGAGGTTTACTCTCAG
CGTAACGCGTTTTTTAATTCTAGTTATTAATAGTAATCAATTACGGGGTCATTAGTTCATA
GCCCATATATGG

H_CXCL6-shRNA2(PGMLV-Puro):

GGACTATCATATGCTTACCGTAACTTGAAAGTATTTCGATTTCTTGGCTTTATATATCTTG
TGGAAAGGACGAGGATCCGAAGAACGGGAAGCAAGTTTGCTCGAGCAAACTTGCTTC
CCGTTCTTCTTTTTTAATTCTAGTTATTAATAGTAATCAATTACGGGGTCATTAGTTCATA
GCCCATATATGG

H_CXCL6-shRNA3(PGMLV-Puro):

GGACTATCATATGCTTACCGTAACTTGAAAGTATTTCGATTTCTTGGCTTTATATATCTTG
TGGAAAGGACGAGGATCCGATTGGTAAACTGCAGGTGTTCTCGAGAACACCTGCAGTT
TACCAATCTTTTTTAATTCTAGTTATTAATAGTAATCAATTACGGGGTCATTAGTTCATAG
CCCATATATGG

siRNA

CXCL6-siRNA-1: AUCAUUUUGGGGUUAAUUCUUGAAUUAACCCCAAAAUGAUCG

CXCL6-siRNA-2: UUUGUUUUCUUGUUUUCACUGGUGAAAACAAGAAAACAAAGC

CXCL6-siRNA-3: AAAUUAGCGAUCAUUUUGGGGCCAAAAUGAUCGCUAAUUUGG

3、Primary antibodies used in this study

Antibody	Company (Cat. No.)
CXCL6	Invitrogen (PA5-115276)
GAPDH	Proteintech (HRP-60004)
PCNA	Abcam (ab92552)
Vimentin	Abcam (ab92547)
N-Cadherin	Abcam (ab76011)
E-Cadherin	Abcam (ab76011)
CK7	Abcam (ab199718)
Ki67	Abcam (ab15580)
CD31	ThermoFisher (RP-104306)
VEGF	Proteintech (19003-1-AP)
HIF-1α	Absin (abs120168)
p-PI3K (Tyr 458)	Abmart (P76365R4)
PI3K	Abmart (T40115)
p-AKT(Ser473)	Abmart (T40067)
p-AKT(Thr308)	Abmart (T40068)
AKT	Abmart (T55561)
p-JAK	Abmart (TA2012)
JAK	Cell Signaling Technology(3344T)
p-STAT3(Y705)	Abmart (T56566)
STAT3	Abmart(T55292)
CXCR1	Absin (abs120403)
CXCR2	Proteintech (19538-1-AP)
CD10	Servicebio (GB121120)
CD45	Servicebio (GB113886)
CD3	Servicebio (GB13014)
CD8	Servicebio (GB115692)
CD68	Servicebio (GB11093)
Foxp3	Servicebio (GB112325)
RAS	Abmart (T56672)
MEK	Abmart (T55168)
p-MEK	Abmart (TA8035)
p-ERK	Abmart (T40072)

ERK	Abmart (T40071)
NE	Abcam (ab131260)
MPO	Abcam (ab208670)
PE-CD45	BioLegend (368509)
FITC-CD16	BioLegend (302005)
APC-cy7-Zombie	BioLegend (423106)
percpcy5.5-CD45	BioLegend (103130)
APC-CD8	BioLegend (100712)
BV421-IFNγ	BioLegend (505830)
PE-cy7-GzmB	BioLegend (372214)

4、Reagents used in this study

**Name**	**Cat no.**	**Supplier**
Gemcitabine	S1714	Selleck
3-Methyladenine	S2767	Selleck
Ruxolitinib	S1378	Selleck
SB225002	S7651	Selleck
Reparixin	S8640	Selleck
Anti-mouse PD-1 (CD279)	BE0146	BioXCell
Rat IgG2a isotype control	BE0089	BioXCell
Cl-amidine	HY-100574A	MCE
TrypLE™ Express Enzyme	12605036	ThermoFisher

Supplementary Tables

Table S1 Clinicopathological features of patients in 'Surgery Cohort' tissue microarray.

Variables	Surgery Cohort	
	N=192	%
Age(y)		
≤50	34	17.71%
>50	158	82.29%
Sex		
Female	67	34.90%
Male	125	65.10%
Child-Pugh		
A	183	95.31%
B	9	4.69%
HBsAg		
Negative	128	66.67%
Positive	64	33.33%
ALT(U/L)		
≤50	172	89.58%
>50	20	10.42%
AFP (ng/mL)		
≤20	176	91.67%
>20	16	8.33%
CA199		
≤34	108	56.25%
>34	84	43.75%
Macrovascular tumor thrombus		
No	171	89.06%
Yes	21	10.94%
TNM		
0 ~ II	152	79.17%
IIIA ~ IIIB	40	20.83%

Abbreviation: ALT, alanine aminotransferase; AFP, alpha-fetoprotein; CA19-9, carbohydrate antigen19-9

Table S2 Clinicopathological features of patients in ‘Conversion therapy cohort’ tissue microarray.

Variables	Conversion Therapy Cohort	
	N=33	%
Age(y)		
≤50	11	33.33%
>50	22	66.67%
Sex		
Female	16	48.48%
Male	17	51.52%
Child-Pugh		
A	29	87.88%
B	4	12.12%
HBsAg		
Negative	23	69.70%
Positive	10	30.30%
ALT(U/L)		
≤50	27	81.82%
>50	6	18.18%
AFP (ng/mL)		
≤20	29	87.88%
>20	4	12.12%
CA199		
≤34	18	54.55%
>34	15	45.45%
Macrovascular tumor thrombus		
No	26	78.79%
Yes	7	21.21%
TNM		
0 ~ II	22	66.67%
IIIA ~ IIIB	11	33.33%

Abbreviation: ALT, alanine aminotransferase; AFP, alpha-fetoprotein; CA19-9, carbohydrate antigen19-9

Table S3 Clinicopathological features of CCA patients in 'Immunotherapy Cohort'.

Variables	Immunotherapy Cohort	
	N=26	%
Age(y)		
≤50	2	7.69%
>50	24	92.31%
Sex		
Female	11	42.31%
Male	15	57.69%
Child-Pugh		
A	19	73.08%
B	7	26.92%
HBsAg		
Negative	23	88.46%
Positive	3	11.54%
ALT(U/L)		
≤50	23	88.46%
>50	3	11.54%
AFP (ng/mL)		
≤20	19	73.08%
>20	7	26.92%
CA19-9		
≤34	12	46.15%
>34	14	53.85%
Macrovascular tumor thrombus		
No	14	53.85%
Yes	12	46.15%
TNM		
0 ~ II	9	34.62%
IIIA ~ IIIB	17	65.38%

Abbreviation: ALT, alanine aminotransferase; AFP, alpha-fetoprotein; CA19-9, carbohydrate antigen19-9

Figure S1

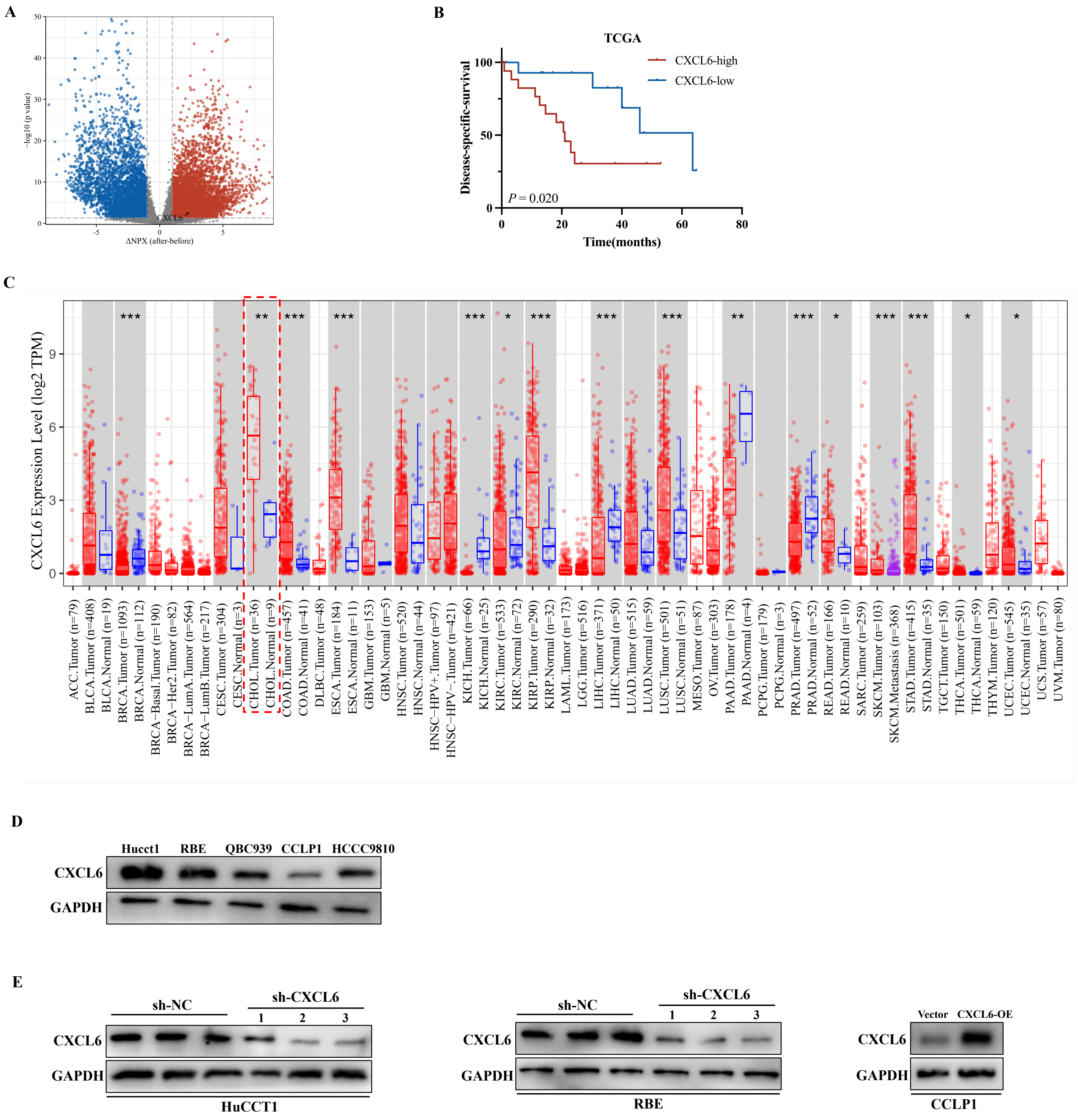
A
-log10 (p value)
ΔNPX (after-before)
CXCL6
B
TCGA
CXCL6-high
CXCL6-low
Disease-specific-survival
P = 0.020
Time(months)
C
CXCL6 Expression Level (log2 TPM)
ACC.Tumor (n=79)
BLCA.Tumor (n=408)
BLCA.Normal (n=19)
BRCA.Tumor (n=1093)
BRCA.Normal (n=112)
BRCA−Basal.Tumor (n=190)
BRCA−Her2.Tumor (n=82)
BRCA−LumA.Tumor (n=564)
BRCA−LumB.Tumor (n=217)
CESC.Tumor (n=304)
CESC.Normal (n=3)
CHOL.Tumor (n=36)
CHOL.Normal (n=9)
COAD.Tumor (n=457)
COAD.Normal (n=41)
DLBC.Tumor (n=48)
ESCA.Tumor (n=184)
ESCA.Normal (n=11)
GBM.Tumor (n=153)
GBM.Normal (n=5)
HNSC.Tumor (n=520)
HNSC.Normal (n=44)
HNSC−HPV+.Tumor (n=97)
HNSC−HPV−.Tumor (n=421)
KICH.Tumor (n=66)
KICH.Normal (n=25)
KIRC.Tumor (n=533)
KIRC.Normal (n=72)
KIRP.Tumor (n=290)
KIRP.Normal (n=32)
LAML.Tumor (n=173)
LGG.Tumor (n=516)
LIHC.Tumor (n=371)
LIHC.Normal (n=50)
LUAD.Tumor (n=515)
LUAD.Normal (n=59)
LUSC.Tumor (n=501)
LUSC.Normal (n=51)
MESO.Tumor (n=87)
OV.Tumor (n=303)
PAAD.Tumor (n=178)
PAAD.Normal (n=4)
PCPG.Tumor (n=179)
PCPG.Normal (n=3)
PRAD.Tumor (n=497)
PRAD.Normal (n=52)
READ.Tumor (n=166)
READ.Normal (n=10)
SARC.Tumor (n=259)
SKCM.Tumor (n=103)
SKCM.Metastasis (n=368)
STAD.Tumor (n=415)
STAD.Normal (n=35)
TGCT.Tumor (n=150)
THCA.Tumor (n=501)
THCA.Normal (n=59)
THYM.Tumor (n=120)
UCEC.Tumor (n=545)
UCEC.Normal (n=35)
UCS.Tumor (n=57)
UVM.Tumor (n=80)
D
Hucct1
RBE
QBC939
CCLP1
HCCC9810
CXCL6
GAPDH
E
sh-NC
sh-CXCL6
1
2
3
CXCL6
GAPDH
HuCCT1
RBE
Vector
CXCL6-OE
CCLP1

Figure S2

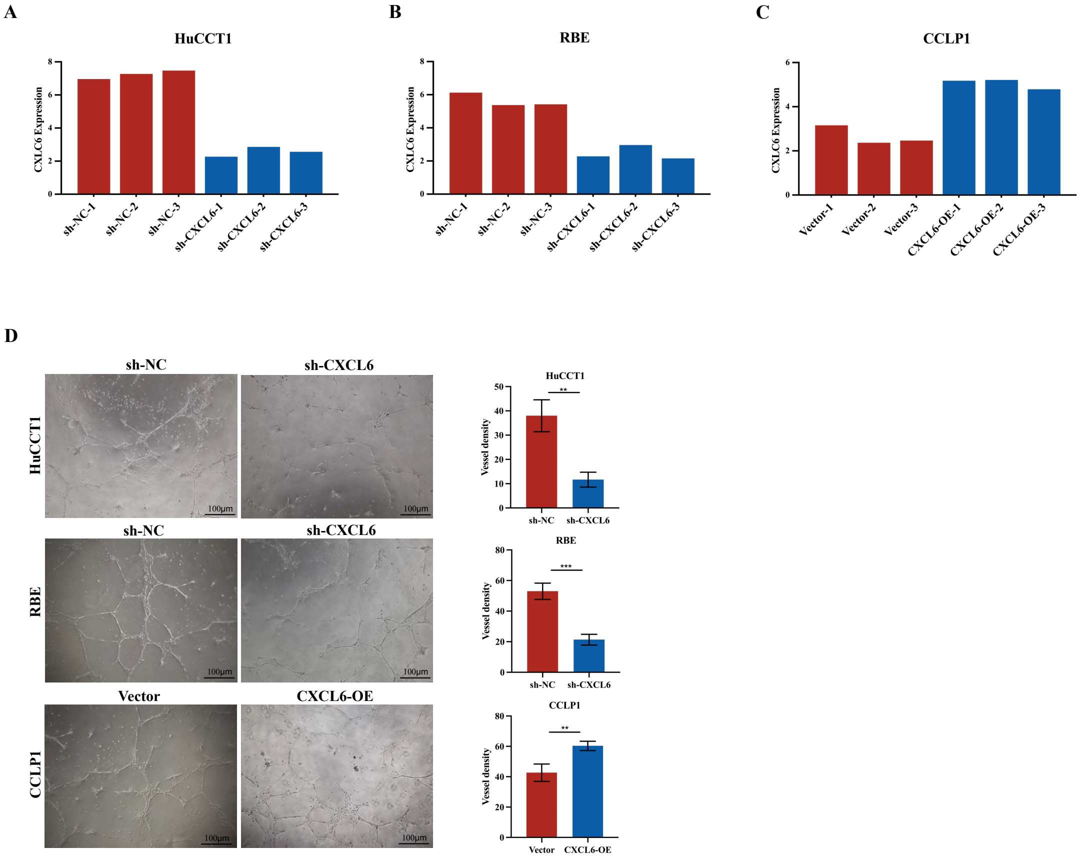

A
HuCCT1
CXLC6 Expression
sh-NC-1
sh-NC-2
sh-NC-3
sh-CXCL6-1
sh-CXCL6-2
sh-CXCL6-3
B
RBE
CXLC6 Expression
sh-NC-1
sh-NC-2
sh-NC-3
sh-CXCL6-1
sh-CXCL6-2
sh-CXCL6-3
C
CCLP1
CXLC6 Expression
Vector-1
Vector-2
Vector-3
CXCL6-OE-1
CXCL6-OE-2
CXCL6-OE-3
D
sh-NC
sh-CXCL6
HuCCT1
RBE
Vector
CXCL6-OE
CCLP1
100μm
Vessel density
**
***
sh-NC
sh-CXCL6
Vector
CXCL6-OE

Figure S3

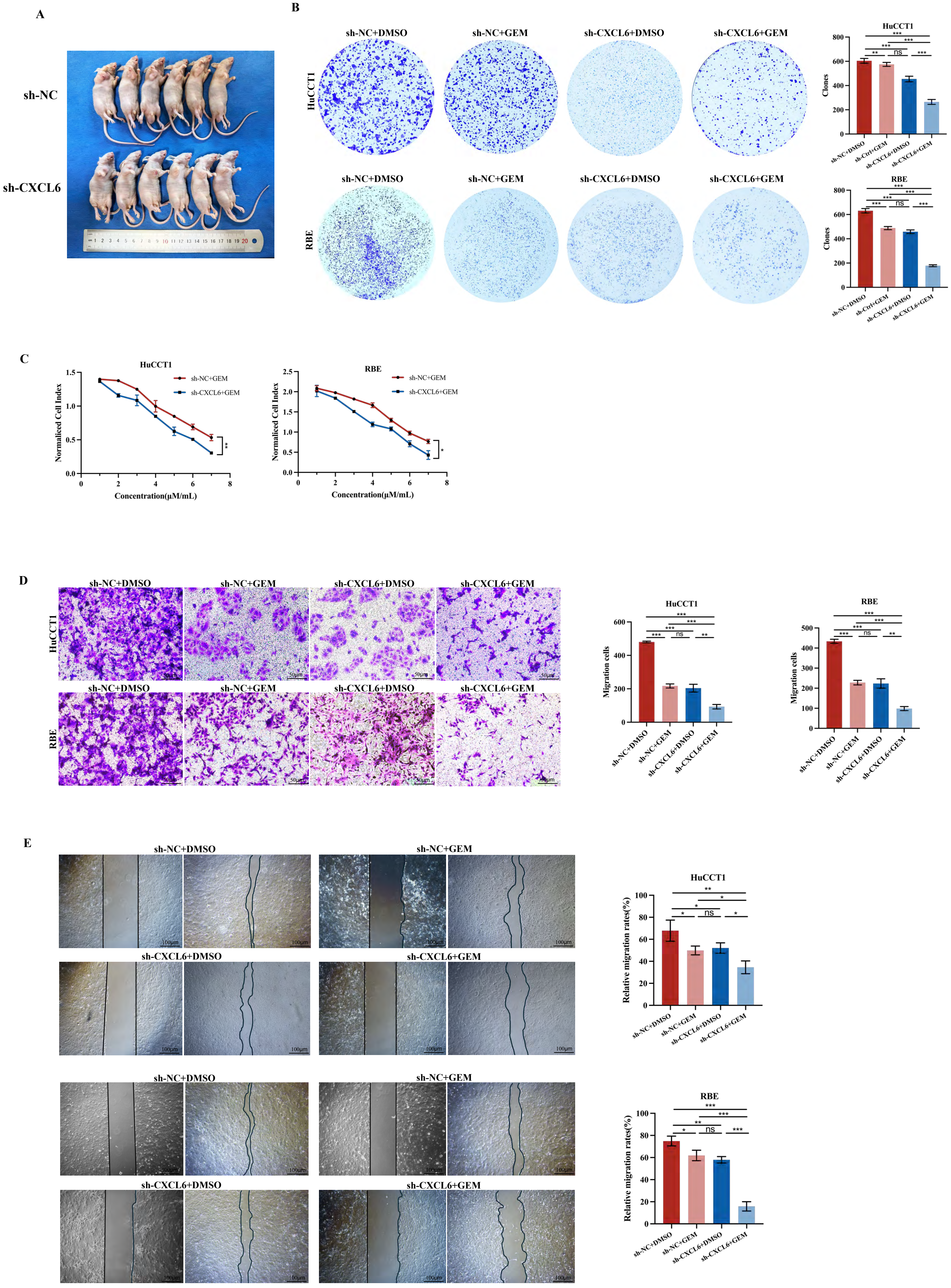
A
sh-NC
sh-CXCL6
B
sh-NC+DMSO
sh-NC+GEM
sh-CXCL6+DMSO
sh-CXCL6+GEM
HuCCT1
RBE
Clones
sh-NC+DMSO
sh-Ctrl+GEM
sh-CXCL6+DMSO
sh-CXCL6+GEM
C
HuCCT1
RBE
sh-NC+GEM
sh-CXCL6+GEM
Normaliced Cell Index
Concentration(μM/mL)
D
Migration cells
sh-NC+GEM
E
Relative migration rates(%)
100μm
50μm
ns

Figure S4

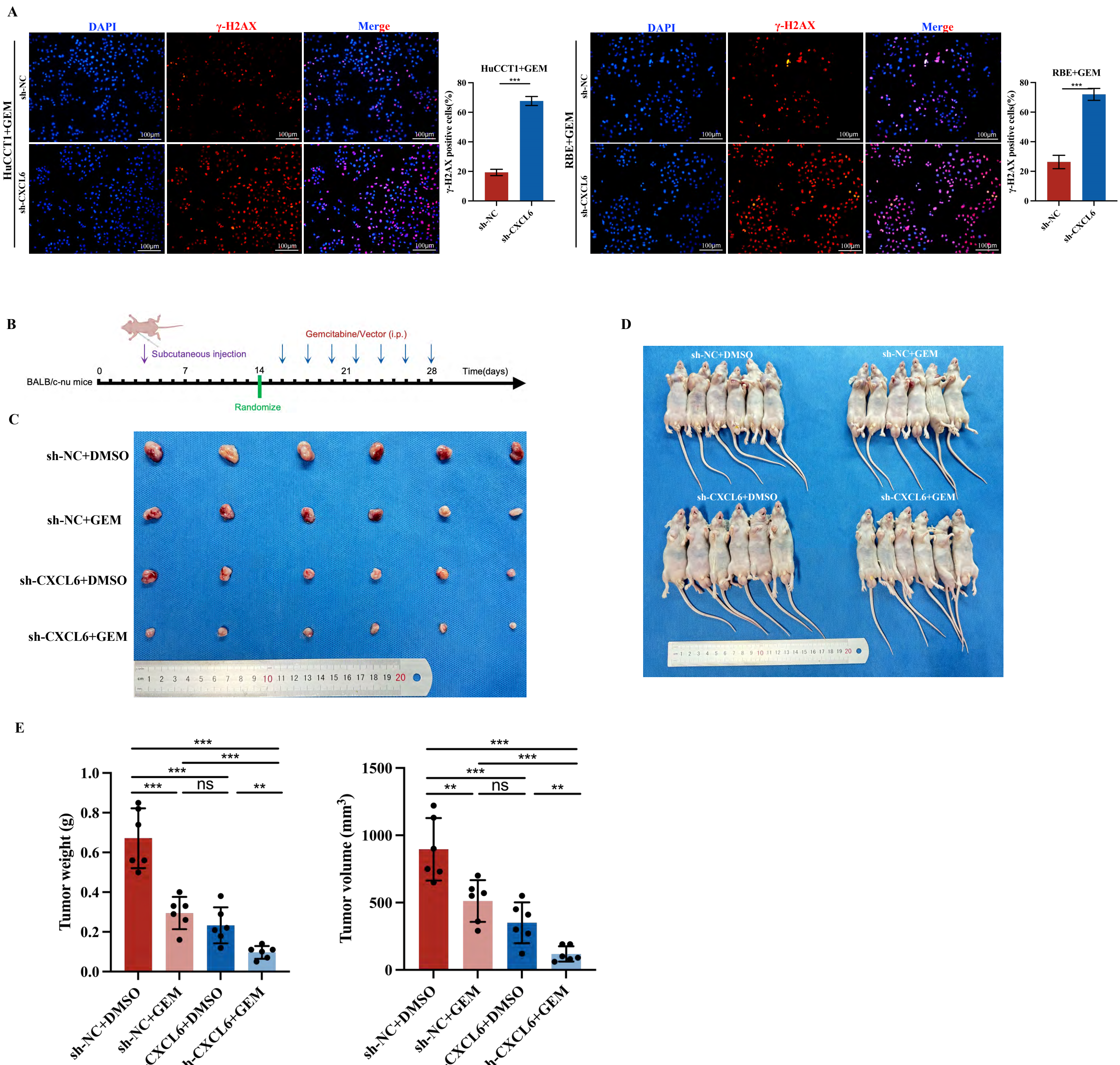
A
DAPI
γ-H2AX
Merge
HuCCT1+GEM
sh-NC
sh-CXCL6
100μm
HuCCT1+GEM
γ-H2AX positive cells(%)
RBE+GEM
RBE+GEM
***
B
Gemcitabine/Vector (i.p.)
Subcutaneous injection
BALB/c-nu mice
0
7
14
21
28
Time(days)
Randomize
C
sh-NC+DMSO
sh-NC+GEM
sh-CXCL6+DMSO
sh-CXCL6+GEM
D
E
Tumor weight (g)
Tumor volume (mm3)
ns
**

Figure S5

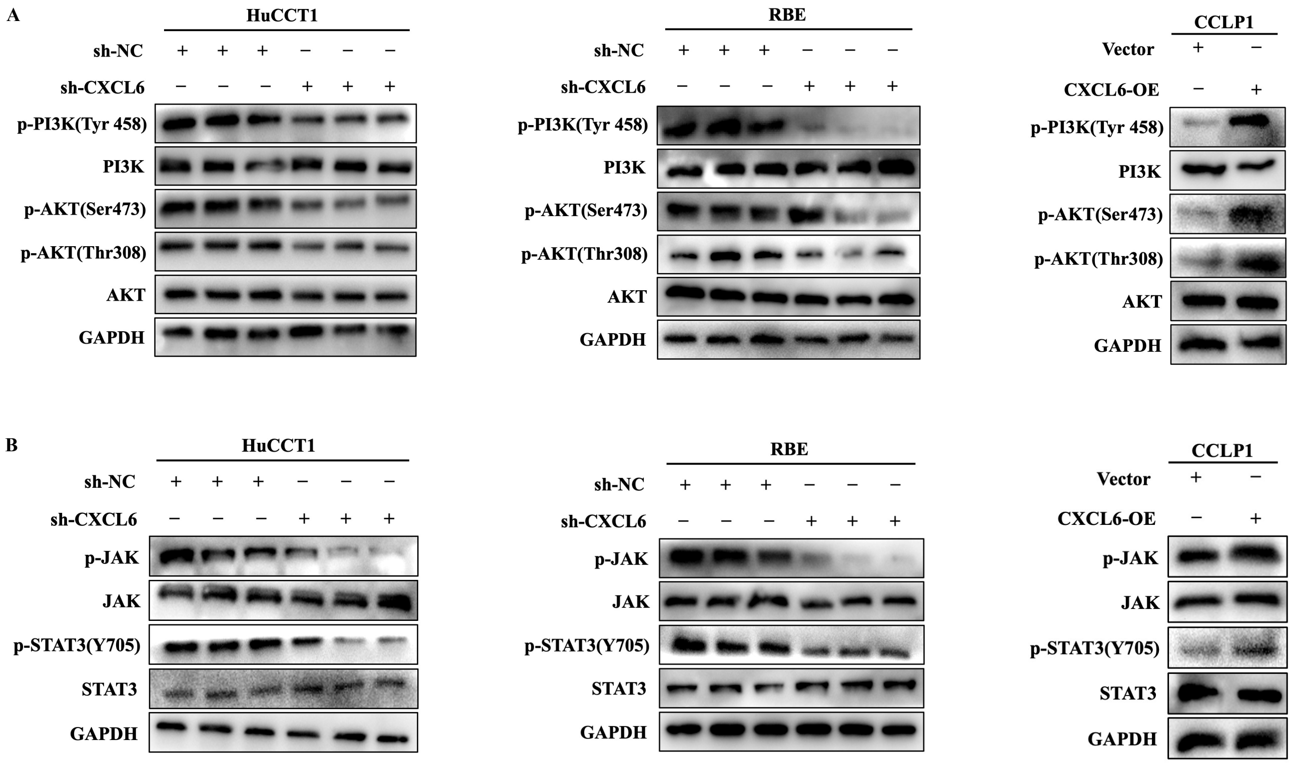

A
HuCCT1
sh-NC + + + − − −
sh-CXCL6 − − − + + +
p-PI3K(Tyr 458)
PI3K
p-AKT(Ser473)
p-AKT(Thr308)
AKT
GAPDH
RBE
sh-NC + + + − − −
sh-CXCL6 − − − + + +
p-PI3K(Tyr 458)
PI3K
p-AKT(Ser473)
p-AKT(Thr308)
AKT
GAPDH
CCLP1
Vector + −
CXCL6-OE − +
p-PI3K(Tyr 458)
PI3K
p-AKT(Ser473)
p-AKT(Thr308)
AKT
GAPDH
B
HuCCT1
sh-NC + + + − − −
sh-CXCL6 − − − + + +
p-JAK
JAK
p-STAT3(Y705)
STAT3
GAPDH
RBE
sh-NC + + + − − −
sh-CXCL6 − − − + + +
p-JAK
JAK
p-STAT3(Y705)
STAT3
GAPDH
CCLP1
Vector + −
CXCL6-OE − +
p-JAK
JAK
p-STAT3(Y705)
STAT3
GAPDH

Figure S6

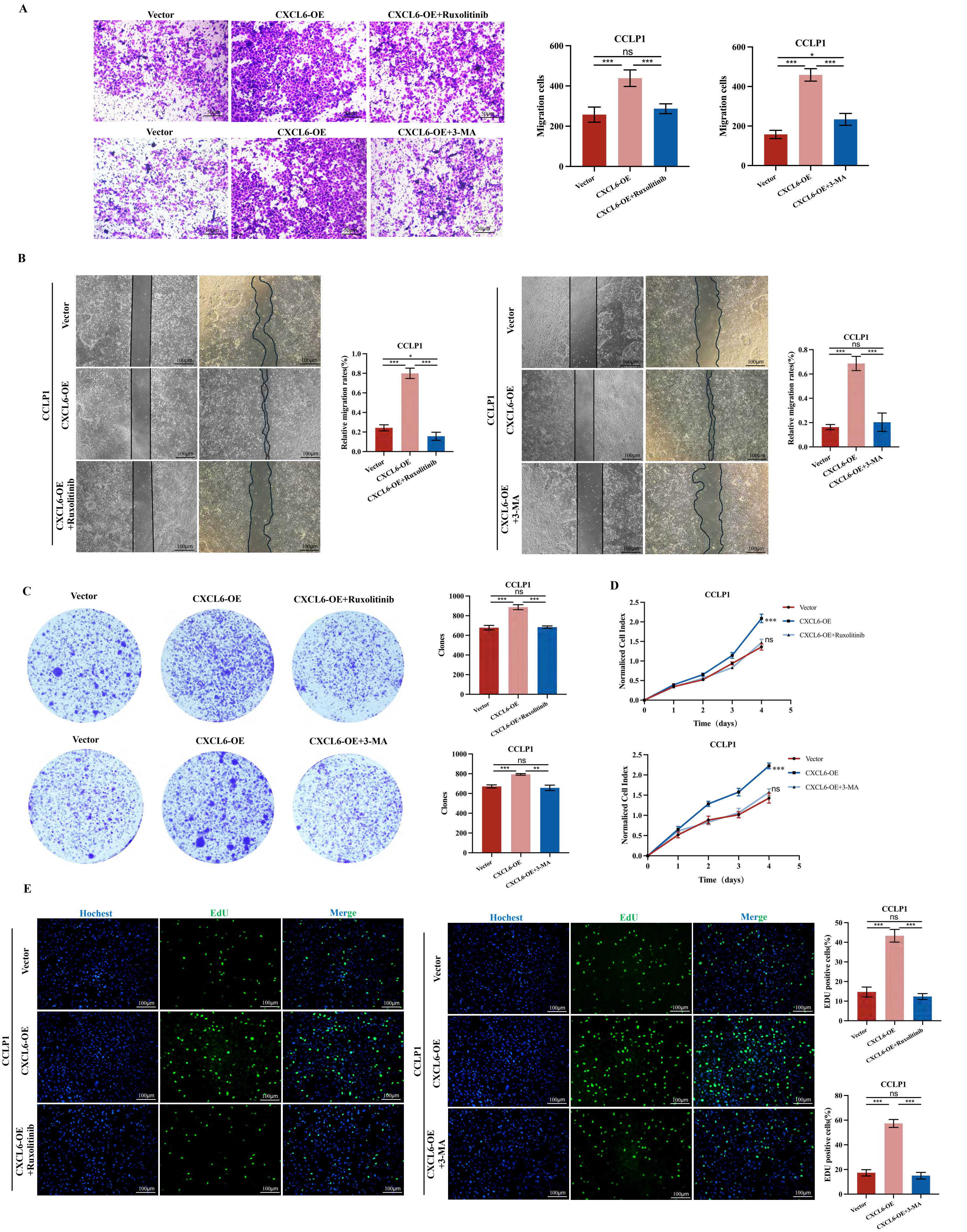

A
Vector
CXCL6-OE
CXCL6-OE+Ruxolitinib
CXCL6-OE+3-MA
CCLP1
Migration cells
B
CCLP1
Vector
CXCL6-OE
CXCL6-OE +Ruxolitinib
CXCL6-OE +3-MA
Relative migration rates(%)
C
Clones
D
Normalized Cell Index
Time (days)
E
Hochest
EdU
Merge
EDU positive cells(%)

Figure S7

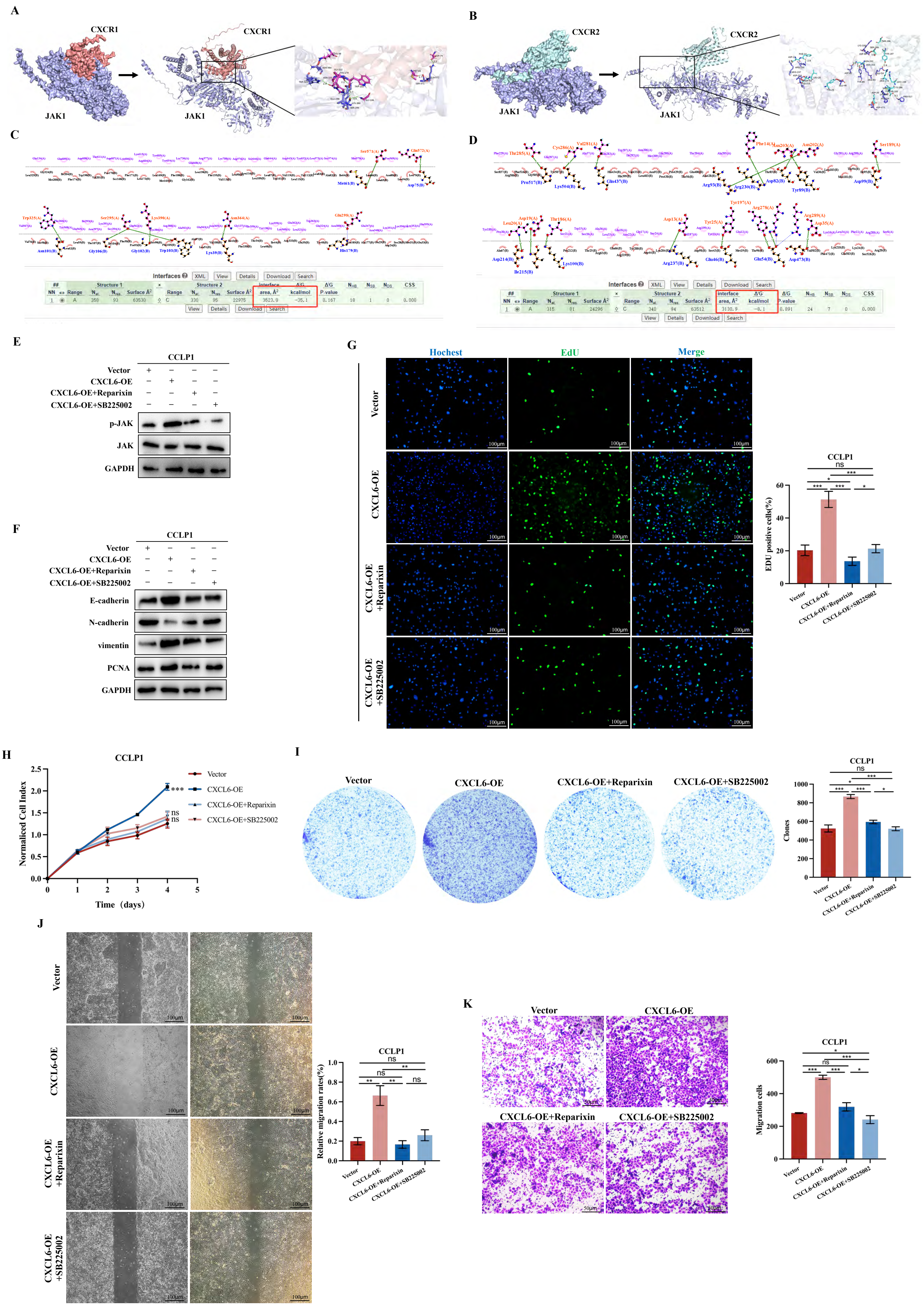
A
CXCR1
JAK1
B
CXCR2
JAK1
C
D
E
CCLP1
Vector
CXCL6-OE
CXCL6-OE+Reparixin
CXCL6-OE+SB225002
p-JAK
JAK
GAPDH
F
CCLP1
E-cadherin
N-cadherin
vimentin
PCNA
GAPDH
G
Hochest
EdU
Merge
Vector
CXCL6-OE
CXCL6-OE +Reparixin
CXCL6-OE +SB225002
100μm
CCLP1
EDU positive cells(%)
H
CCLP1
Normalized Cell Index
Time (days)
I
Vector
CXCL6-OE
CXCL6-OE+Reparixin
CXCL6-OE+SB225002
CCLP1
Clones
J
Relative migration rates(%)
K
Migration cells
50μm

Figure S8

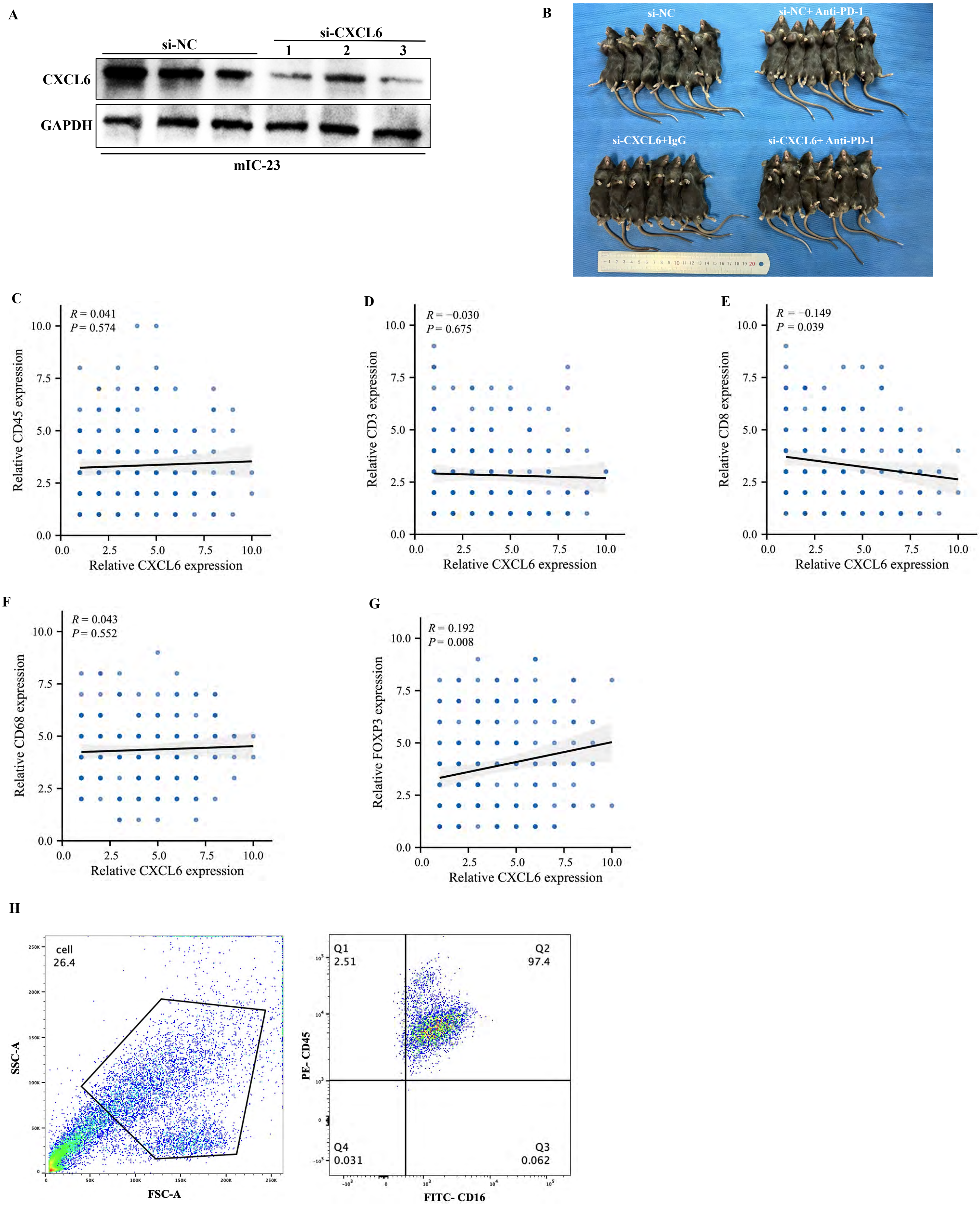

A
si-NC
si-CXCL6
1
2
3
CXCL6
GAPDH
mIC-23
B
si-NC
si-NC+ Anti-PD-1
si-CXCL6+IgG
si-CXCL6+ Anti-PD-1
C
R = 0.041
P = 0.574
Relative CD45 expression
Relative CXCL6 expression
D
R = −0.030
P = 0.675
Relative CD3 expression
Relative CXCL6 expression
E
R = −0.149
P = 0.039
Relative CD8 expression
Relative CXCL6 expression
F
R = 0.043
P = 0.552
Relative CD68 expression
Relative CXCL6 expression
G
R = 0.192
P = 0.008
Relative FOXP3 expression
Relative CXCL6 expression
H
cell
26.4
SSC-A
FSC-A
Q1
2.51
Q2
97.4
Q4
0.031
Q3
0.062
PE- CD45
FITC- CD16

Figure S9

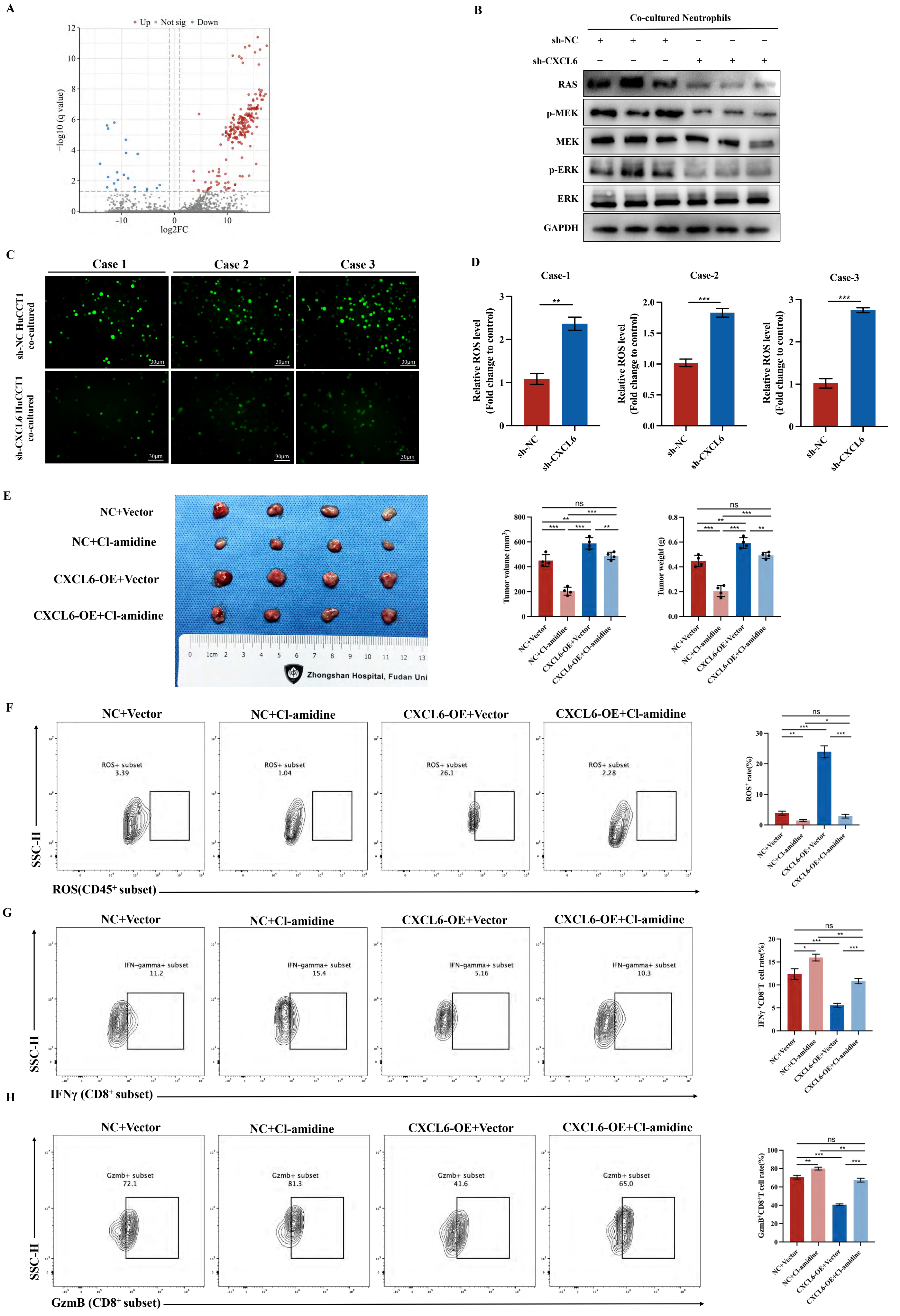
A
Up
Not sig
Down
−log10 (q value)
log2FC
B
Co-cultured Neutrophils
sh-NC
sh-CXCL6
RAS
p-MEK
MEK
p-ERK
ERK
GAPDH
C
Case 1
Case 2
Case 3
sh-NC HuCCT1 co-cultured
sh-CXCL6 HuCCT1 co-cultured
30μm
D
Case-1
Case-2
Case-3
Relative ROS level (Fold change to control)
sh-NC
sh-CXCL6
E
NC+Vector
NC+Cl-amidine
CXCL6-OE+Vector
CXCL6-OE+Cl-amidine
Zhongshan Hospital, Fudan Uni
Tumor volume (mm³)
Tumor weight (g)
ns
F
ROS+ subset
3.39
1.04
26.1
2.28
SSC-H
ROS(CD45⁺ subset)
ROS⁺ rate(%)
G
IFN-gamma+ subset
11.2
15.4
5.16
10.3
IFNγ (CD8⁺ subset)
IFNγ⁺CD8⁺T cell rate(%)
H
Gzmb+ subset
72.1
81.3
41.6
65.0
GzmB (CD8⁺ subset)
GzmB⁺CD8⁺T cell rate(%)

Figure S10

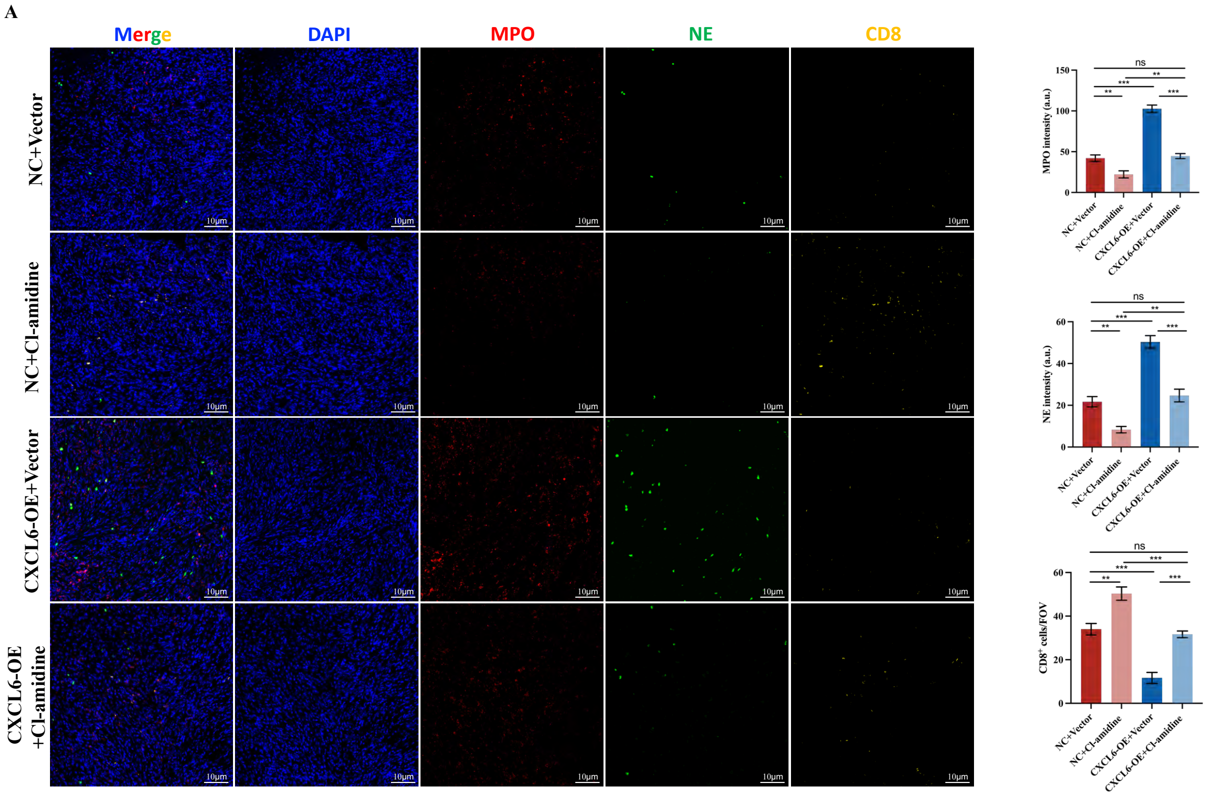
A
Merge
DAPI
MPO
NE
CD8
NC+Vector
NC+Cl-amidine
CXCL6-OE+Vector
CXCL6-OE
+Cl-amidine
10μm
MPO intensity (a.u.)
NE intensity (a.u.)
CD8+ cells/FOV
ns
**
***
NC+Vector
NC+Cl-amidine
CXCL6-OE+Vector
CXCL6-OE+Cl-amidine

Figure legends

Figure S1. CXCL6 is upregulated in CCA tumor tissue and relates to poor prognosis.

(A) Volcano plot of differential gene expression between CCA tumor tissues and paired non-tumor tissues, based on TCGA. (B) High *CXCL6* expression is associated with poorer DSS, based on TCGA. (C) *CXCL6* expression levels in tumor tissues and paired non-tumor tissues in multiple cancers, based on TCGA. (D) Western blot analysis of CXCL6 expression in CCA cell lines. (E) Western blot analysis of CXCL6 silencing and overexpression efficiency.

Figure S2. CXCL6 level in conditioned medium of transfected CCA.

(A- B) Elisa confirmation of CXCL6 level in sh-CXCL6, sh-NC HuCCT1 and RBE cells. (C) Elisa confirmation of CXCL6 level in Vector, CXCL6-OE CCLP1 cells. (D) HUVEC tube formation assay showed that CXCL6 is pro-angiogenic.

Figure S3. CXCL6 silencing enhances GEM treatment efficacy in CCA in vitro.

(A) Representative images of BALB/c nude mice with subcutaneously established tumors. (B,C) Colony formation and CCK-8 assays of GEM-treated HuCCT1 and RBE CCA cells with or without *CXCL6* knockdown. (D,E) Transwell and wound healing assays of CCA cells with or without *CXCL6* knockdown, treated with GEM or vehicle control (DMSO).

Figure S4. CXCL6 silencing enhances GEM treatment efficacy in CCA in vivo.

(A) γ-H2AX staining was used to detect DNA damage after GEM treatment.(B) Experimental design and dosing regimen for the nude mouse subcutaneous tumors. (C - E) Representative images and measurements of GEM-treated nude mice with subcutaneously established tumors, treated with GEM.

Figure S5. Western blot confirmation of pathways suppression by CXCL6 silencing.

(A) Western blot analysis of PI3K-AKT pathway regulation after CXCL6 knockdown or overexpression. (B) Western blot analysis of JAK-STAT pathway regulation after CXCL6 knockdown or overexpression.

Figure S6. Ruxolitinib and 3-MA inhibit the effect of CXCL6 in promoting CCA cells progression.

(A,B) Transwell and wound healing assays showed that both inhibitors attenuated the effect of *CXCL6* overexpression on CCA migration. (C,D) Colony formation and CCK-8 assays revealed that ruxolitinib and 3-MA both inhibited the pro-proliferative effect of *CXCL6* overexpression on CCA cells. (E) EdU incorporation assays revealed that ruxolitinib and 3-MA both inhibit the increased proliferation seen with CXCL6 overexpression.

Figure S7. CXCL6 functions through CXCR1/2-JAK activation.

(A,B) Models of CXCR1/JAK1 and CXCR2/JAK1 binding from molecular docking. (C,D) Detailed results from molecular docking between CXCR1/JAK1 and CXCR2/JAK1. (E) Reparixin (CXCR1 antagonist) and SB225002 (CXCR2 antagonist) inhibited CXCL6-associated phosphorylation of JAK. (F) Both compounds also dowregulated expression of E-cadherin, N-cadherin, vimentin, and PCNA in CXCL6-overexpressing CCLP1 cells. (G–I) EdU incorporation, CCK-8, and colony formation assays showed that reparixin and SB225002 both attenuate the proliferation of CCLP1 cells induced by CXCL6 overexpression. (J,K) Wound healing and transwell assays indicated that the same compounds can inhibit migration of CXCL6-overexpressing CCLP1 cells.

Figure S8. Correlation between CXCL6 and common immune cell markers in IHC staining.

(A) Western blot analysis of si-RNA transfection efficiency in mIC-23 cell line. (B) Representative images of C57BL/6J mice with subcutaneously established tumors. (C–G) Linear regression analysis between CXCL6 and CD45, CD3, CD8, CD68, and FOXP3 expression in the 'Surgery cohort' TMA. (H) Flow cytometry was applied to confirm isolation of neutrophils.

Figure S9. Confirmation of NETs formation and its relationship with $CD8^{+}$T cell infiltration.

(A) Volcano plot of differently expressed genes in neutrophils according to RNA-Seq data. (B) Western blot assay to assess RAS/MAPK pathway activation. (C–D) ROS staining of TANs cocultured with sh-NC or sh-CXCL6 HuCCT1 cells and statistical analysis. (E) Representative images and statistical comparison of tumors in rescue experiment. (F–H) Flow cytometry of ROS, $IFN\gamma^{+}CD8^{+}$ and $GzmB^{+}CD8^{+}$ in rescue experiment.

Figure S10. IHC staining of NETs formation and $CD8^{+}$T cell infiltration level.

(A) IHC staining and statistical analysis of MPO, NE and CD8 in based on tissues from rescue experiment.
